# Efficacy of dual intracerebroventricular and intravitreal *CLN5* gene therapy in sheep prompts the first clinical trial to treat CLN5 Batten disease

**DOI:** 10.3389/fphar.2023.1212235

**Published:** 2023-10-24

**Authors:** Samantha J. Murray, Martin P. Wellby, Graham K. Barrell, Katharina N. Russell, Ashley R. Deane, John R. Wynyard, Steven J. Gray, David N. Palmer, Nadia L. Mitchell

**Affiliations:** ^1^ Faculty of Agriculture and Life Sciences, Lincoln University, Lincoln, New Zealand; ^2^ Department of Pediatrics, University of Texas Southwestern Medical Center, Dallas, TX, United States; ^3^ Department of Radiology, University of Otago, Christchurch, New Zealand

**Keywords:** neuronal ceroid lipofuscinosis, neurodegenerative disease, gene therapy, adeno-associated virus, intracerebroventricular, intravitreal, sheep

## Abstract

Mutations in the *CLN5* gene cause the fatal, pediatric, neurodegenerative disease CLN5 neuronal ceroid lipofuscinosis. Affected children suffer progressive neuronal loss, visual failure and premature death. Presently there is no treatment. This study evaluated dual intracerebroventricular (ICV) and intravitreal (IVT) administration of a self-complementary adeno-associated viral vector encoding ovine *CLN5* (scAAV9/oCLN5) into CLN5 affected sheep (CLN5^−/−^) at various disease stages. CLN5 disease progression was slowed in pre-symptomatic sheep who received a moderate dose of scAAV9/oCLN5, whilst a higher ICV dose treatment in early and advanced symptomatic animals delayed or halted disease progression. Intracranial (brain) volume loss was attenuated in all treatment cohorts, and visual function was also sustained in both the early and advanced symptomatic treated sheep over the 24-month duration of the study. Robust CLN5 protein expression was detected throughout the brain and spinal cord, and improvements in central nervous system and retinal disease correlates were observed. These findings hold translational promise for extending and improving the quality of life in both pre-symptomatic and symptomatic CLN5 patients, and prompted the initiation of the first in-human Phase I/II clinical trial testing ICV/IVT administration of scAAV9 encoding human *CLN5* (https://clinicaltrials.gov/; NCT05228145).

## 1 Introduction

The neuronal ceroid lipofuscinoses (NCLs), also known as Batten disease, are a group of fatal neurodegenerative diseases, primarily affecting children; each form arising from mutations in any one of 13 *CLN* genes. Although more than 58 *CLN5* mutations result in a diverse range of age at symptom onset (infantile to early adult) and pathological and clinical presentations (Basak et al., 2021), initial symptoms that often appear around 5 years of age typically include developmental regression, seizure, ataxia, vision loss, and intellectual disability. Neuropathological features of CLN5 disease include cortical and retinal atrophy, astrocytosis, and accumulation of fluorescent lysosomal storage material (Autti et al., 1992; Tyynelä et al., 1997; Holmberg et al., 2000; Tyynelä et al., 2004; Xin et al., 2010). As with presentation and onset, life expectancy also varies with survival ranging from 10 to 30 years of age.

At present, there is no treatment for CLN5 Batten disease. As a monogenic disease, it is attractive for gene therapy approaches. Moreover, for soluble lysosomal proteins, like CLN5, genetic correction of a small subset of neural cells is predicted to be sufficient to provide clinical benefit, as transduced cells can secrete CLN5 protein for uptake by neighboring protein-deficient cells (Sands and Davidson, 2006).

Naturally occurring *CLN5* mutations have been identified in many species, including New Zealand Borderdale sheep (Jolly et al., 2002; Frugier et al., 2008). Affected sheep can be classified as CLN5-deficient (CLN5^−/−^) as the causative *CLN5* splice site mutation results in a premature stop codon and degradation of the mutant *CLN5* mRNA by nonsense-mediated decay so these animals express no, or very low levels, of truncated CLN5 protein. Prior studies in this model demonstrated the potential efficacy of *CLN5* gene therapy administered via both intracerebroventricular (ICV) and intraparenchymal (IP) injection in pre-symptomatic animals as well as those with early-stage disease (Mitchell et al., 2018). Development of neurological CLN5 disease was delayed; however, although vision failure was delayed compared with untreated CLN5 sheep, the treated sheep still ultimately went blind. Furthermore, disease progression and brain atrophy were attenuated in sheep with early-stage or even advanced-stage disease treated with ICV delivery of recombinant self-complementary AAV9 vectors expressing codon-optimized ovine *CLN5* (scAAV9/oCLN5) (Mitchell et al., 2018) (Mitchell et al., 2023a, manuscript co-submitted). In a separate study, intravitreal (IVT) administration of the same gene therapy vector ameliorated retinal function and structure in the treated eyes while the untreated eyes showed stereotypical loss of function and disease-associated pathology (Murray et al., 2021). However, as treated sheep did not receive brain-directed therapy, they still lost their vision, likely due to cortical blindness, and succumbed to the disease at 18 months of age.

Although efficacy outcomes from the previous ICV-only and IVT-only scAAV9/oCLN5 gene therapy studies were promising, neither route of administration alone was sufficient to preserve both neurological and visual function. As such, the present study was undertaken to evaluate the impact of combined ICV and IVT administration of scAAV9/oCLN5 in affected sheep. Given the progressive nature of CLN5 Batten disease, early intervention is predicted to be the most efficacious; however, most human cases are diagnosed when patients are already symptomatic. Therefore, the effect of combined ICV and IVT scAAV9/oCLN5 administration was assessed at three previously identified disease stages–pre-symptomatic (3 months of age), early symptomatic (6 months of age), and advanced symptomatic (9 months of age) (Mitchell et al., 2023b). All sheep received the same IVT dose and pre-symptomatic sheep received the previously efficacious ICV dose (Mitchell et al., 2023a). Higher ICV doses were delivered to early and advanced symptomatic sheep in an effort to maximize efficacy. The data from these cohorts provide proof-of-concept for the gene therapy product, delivery route, therapeutic window with respect to disease state and allow evaluation of the long-term safety of the therapy and provide useful translational data regarding effective dose.

## 2 Materials and methods

### 2.1 Animals

Borderdale sheep were genetically diagnosed at birth as either clinically healthy CLN5 heterozygous (CLN5^+/−^) or CLN5 affected (CLN5^−/−^) as previously described (Frugier et al., 2008). They were maintained at Lincoln University under NIH guidelines for the Care and Use of Animals in Research and the NZ Animal Welfare Act (1999). The animal studies were reviewed and approved by the Lincoln University Animal Ethics and Institutional Biosafety Committees.

Supplementary Table S1 summarizes the study design. Briefly, nine CLN5^-*/-*^ ewes received combination bilateral intracerebroventricular (ICV) and unilateral intravitreal (IVT) administration of scAAV9/oCLN5 at three different disease stages: pre-symptomatic 3 months (*n*=3), early symptomatic 6 months (*n*=3), or advanced symptomatic 9 months (*n*=3).

Concurrent age-matched CLN5^+/−^ (*n* = 3) and CLN5^−/−^ (*n* = 4) ewes acted as untreated clinically normal and affected controls, respectively. Data from these animals were pooled for the neurological examinations and magnetic resonance imaging (MRI) analyses; historic controls were used for the remainder of the in-life assessments and neuropathological analyses.

### 2.2 Vector and administration

scAAV9/oCLN5 is a recombinant, self-complementary adeno-associated virus serotype 9 (AAV9) encoding a codon-optimized ovine *CLN5* transgene (oCLN5opt) driven by the chicken beta actin (CBh) promoter, produced by the University of North Carolina Gene Therapy Center Vector Core (NC, United States) as previously described (Mitchell et al., 2018). Vectors were formulated in 350 mM PBS containing 5% sorbitol, and titers determined by qualified droplet digital PCR.

Affected CLN5^*−/−*^ sheep (*n* = 3 per treatment age) were administered scAAV9/oCLN5 vector via concurrent ICV and IVT injection. For ICV delivery, stereotactic surgical procedures were used as described previously (Linterman et al., 2011; Mitchell et al., 2018). Bilateral ICV injections were performed in a volume of 400 µL per hemisphere (total volume 800 µL), delivered at a rate of 100 μL/min. Pre-symptomatic 3-month-old CLN5^−/−^ sheep received a moderate dose (MD; 2.9 × 10^11^ viral genomes (vg)) via ICV delivery into the cerebral lateral ventricles, whilst early symptomatic sheep treated at 6 months or advanced symptomatic sheep treated at 9 months of age received a high-dose (HD; 3.3 × 10^12^ vg).

After ICV delivery, anaesthetized sheep were repositioned for IVT administration as previously described (Murray et al., 2021). A total dose of 6.5 × 10^10^ vg scAAV9/oCLN5 was injected in a volume of 100 µL into the vitreous humor of the left eye. The right eye served as an internal control and was not treated. All ICV/IVT treated animals were sacrificed at a pre-determined timepoint (24 months of age) for post-mortem analysis.

### 2.3 Neurological examination

General physical health and neurological clinical assessments were performed monthly on the treated CLN5^−/−^ sheep and age-matched cohorts of healthy CLN5^+/−^ and untreated CLN5^−/−^ sheep by two independent blinded investigators. Scores for each of the ten parameters in the ovine Batten disease rating scale (oBDRS) ranged from 4 to 0 (normal to abnormal), giving a total score of 40 (Mitchell et al., 2018). Cranial nerve function, mentation, gait, head carriage and postural traits, as well as manifest tremor or seizure onset, were reported. Live weight data and body condition scores were also collected.

### 2.4 Visual assessment

As all treated CLN5^−/−^ sheep received IVT injections, the visual domain of the oBDRS was particularly exploited. Visual acuity was assessed through physical examination and in the field. Sheep were graded from 4 to 0 based on the following ten traits: head tilt, response to shadows, visual tracking, menace response, dazzle response, corneal/palpebral reflex, pupillary light reflex, funduscopic changes, electroretinography response, and hitting objects (Supplementary Table S2). In the event of a discrepancy between traits, sheep received the lowest possible grading. The only exception was when a sheep exhibited only a single reduced menace response, dazzle response, corneal reflex or pupillary light reflex (in one or both eyes) but no other visual deficits. In this situation, the sheep would score 3.

### 2.5 Maze testing

Visual and cognitive decline was assessed monthly in a previously described closed-field maze under daytime photopic light (Mitchell et al., 2018). Treated CLN5^−/−^ sheep and age-matched cohorts of healthy CLN5^+/−^ and untreated CLN5^−/−^ sheep individually negotiated the maze five times on each testing day to join conspecifics in the end pen. Sheep were allowed 2 min to complete the maze and the time taken to traverse was recorded. Testing continued monthly until sheep were unable to complete the maze.

### 2.6 Electroretinography

Mixed response ERG recordings were obtained from each eye of treated sheep as previously described (Murray et al., 2021). Dark adapted b-wave amplitudes were compared with historical data from healthy CLN5^+/−^ (*n =* 6) and untreated CLN5^−/−^ sheep (*n =* 6) (Russell et al., 2021).

### 2.7 Quantitative analysis of brain atrophy

Computed tomography (CT) scans were performed on treated sheep every two to 4 months as previously described (Russell et al., 2018). Helical slices were obtained at 1 mm intervals, 120 kV, 100 mA, 2 s rotation time on a GE HD750 CT scanner (GE Healthcare, Hyugo, Japan). Three-dimensional (3D) modelling and intracranial volumetrics were performed using the 3D slicer 4.3.1 freeware (http://www.slicer.org; RRID:SCR_005619) (Federov et al., 2012; Russell et al., 2018).

### 2.8 Immunohistochemistry

Sagittal sheep brain sections were obtained as previously described (Oswald et al., 2005). In brief, sections were blocked, 30 min with 1% H_2_O_2_ in PBS (pH 7.4) containing 0.3% Triton X-100 (PBST) and then 1 h in 15% normal goat serum (NGS) in PBST prior to overnight incubation in primary antibody diluted in 10% NGS in PBST at 4°C. The following primary antibodies were used: rabbit anti-cow glial fibrillary acidic protein (GFAP, 1:5000; Agilent Cat# Z0334, RRID:AB_10013382) to detect activated astrocytes, a biotinylated form of the α-D-galactose specific isolectin I-B4 from *Griffonia simplicifolia* (GSB4, 1:500; Vector Laboratories Cat# B-1205, RRID:AB_2314661) for microglia and rabbit anti-sheep CLN5 (1:500; Viraquest Inc. Cat# R19122) for CLN5 protein expression. A biotinylated goat anti-rabbit IgG secondary antibody (1:1000, Sigma-Aldrich Cat# B7389, RRID:AB_258613) was applied for 4 h at room temperature, followed by ExtrAvidin peroxidase (1:1000, Sigma-Aldrich Cat# E2886, RRID:AB_2620165) for 4 h. Staining was visualized by incubation in 0.5 mg/mL 3, 3′-diaminobenzadine (DAB; Sigma-Aldrich Cat# D5637) and 0.01% H_2_O_2_ in PBS. Sections were mounted in a solution of 0.5% gelatine and 0.05% chromium potassium sulphate on glass slides, air-dried, dehydrated in 100% ethanol, cleared in xylene and coverslips mounted with DPX (BDH Chemicals, Poole, England).

Adjacent sections were processed through a standard cresyl violet Nissl stain to detect neuronal cytoarchitecture (Oswald et al., 2005). A parallel set of unstained sections was mounted, air-dried, and coverslipped with glycerol for fluorescent storage body analysis. Historical 50 µm brain and spinal cord sections from healthy control CLN5^+/−^ (*n* = 3) and diseased CLN5^−/−^ (*n* = 3) sheep were included in all staining runs as negative and positive controls.

### 2.9 Retinal histology

Eye globes were enucleated at the time of death, separated into anterior and posterior segments, fixed in 10% formalin, 2 h, and sent to Gribbles Veterinary Pathology (Christchurch, New Zealand) for post-fixation in Bouin’s solution (Sigma-Aldrich Cat# HT10132), for 4 h followed by wax embedding. Retinal paraffin sections were cut at 3 μm, mounted and a subset stained with Hematoxylin and Eosin (H + E) histological stain by Gribbles Veterinary Pathology for analysis of retinal thickness. A parallel subset of unstained retinal sections was coverslipped in glycerol to assess fluorescent storage body accumulation.

### 2.10 Microscopy

Digital images of CLN5, GFAP, GSB4, Nissl-stained and unstained brain sections were obtained with a Nikon Digital Sight DSFi1 camera attached to a Nikon Eclipse 50i model microscope (Nikon Instruments Inc., Tokyo, Japan) utilizing NIS-Elements Software (Nikon Instruments; RRID:SCR_014329).

CLN5-positive somata and fibres were semi-quantified (from zero to 100 CLN5-positive somata/0.57 mm^2^ field) in 20 fields for 11 distinct brain regions within each treated sheep hemisphere and 25 fields for three spinal cord regions (cervical, thoracic and lumbar). At least 25 thickness measurements were taken through three different cortical regions on Nissl-stained sections (Oswald et al., 2005). A set of 10 non-overlapping red-green-blue (RGB) images were captured at each sampling site per hemisphere for each immunostain, or with a 450–490 excitation/510 emission filter set for observation of fluorescent storage bodies.

Images were analyzed with ImageJ software (version 1.52p; National Institutes of Health (NIH), Bethesda, MD, United States; RRID:SCR_003070). Red bandwidth filters were applied, and a constant threshold value was used for each image set such that positively stained structures with low reactivity were still selected, but not background staining in regions of high reactivity. Results are presented as the percentage of immunoreactivity or fluorescence per sampled area.

Twenty total retinal thickness measurements per eye were taken from the surface of the nerve fiber layer (NFL) to the base of the retinal pigment epithelium (RPE) in both the central retina (within 5 mm of the optic nerve head) and the peripheral retina (10–20 mm from the optic nerve head). The number of photoreceptor nuclei were counted in 20 vertical columns through the outer nuclear layer of hematoxylin and eosin-stained retinal sections for each eye. In addition, ten images from the central retina were collected from unstained sections for each eye and thresholding analysis on ImageJ was used to determine the percentage of fluorescence per sampled area. The RPE and photoreceptor inner segment/outer segment layers were excluded from storage body analysis due to the endogenous fluorescence present in these cellular layers in the normal sheep eye.

### 2.11 Statistical analysis

All statistical analyses of neuropathological data were performed on GraphPad Prism software (v 9,0.0, GraphPad, La Jolla, CA, United States; RRID:SCR_002798). For most analyses, results were reported as mean ± the standard errors of the mean (SEM). Differences were considered as statistically significant where *p* < 0.05.

For the longitudinal ERG study, b-wave amplitudes were measured for each eye using the provided software (Eickemeyer). Animals were grouped by treatment and the repeated measurements were allocated into age-groups. ERGs were compared between treated and untreated eyes and over time using linear mixed regression models, with the lme4 package in R studio (v4.0.5; RRID:SCR_015654) (Bates et al., 2015; R Core Team, 2021). For each genotype, ERG was modelled independently as a function of age, treatment, and age-by-treatment interaction, with a varying intercept and slope for each sheep.

For the neuropathological studies, means (% area stained, cortical and retinal thickness, ONL count) and the corresponding SEM were computed for each brain region for each animal. These are presented in Supplementary Tables S3–S9. The means were used in a one-way Brown-Forsythe and Welch ANOVA followed by multiple unpaired t-tests assuming unequal variances and using a false discovery approach with the two-stage linear step-up procedure of Benjamini, Krieger and Tekutieli with a Q = 0.05 to test each region separately for differences between control, treated and untreated CLN5^−/−^ cohorts.

## 3 Results

### 3.1 *In vivo* vector delivery

Bilateral ICV and unilateral IVT delivery was possible for all animals in the study. The treatment was well tolerated, and no safety concerns were identified with the delivery method or dose. In-life data were collected over the study before treated sheep were sacrificed for post-mortem analysis at a pre-determined timepoint of 24 months of age.

### 3.2 Live weight

All the ICV/IVT treated animals showed steady weight gains over their first year, peaking at 16 months of age (Supplementary Figure S1). Two pre-symptomatic (1124/18, 1128/18) and two early symptomatic (1151/18, 1157/18) treated sheep reached healthy adult weights of 65–69 kg. However, the weights of advanced symptomatic treated sheep tracked closely with untreated CLN5^−/−^ controls, and one pre-symptomatic treated sheep (1102/18) was considerably lighter than the average CLN5^−/−^ sheep over her lifetime.

### 3.3 Clinical signs and neurobehaviour

**FIGURE 1:**
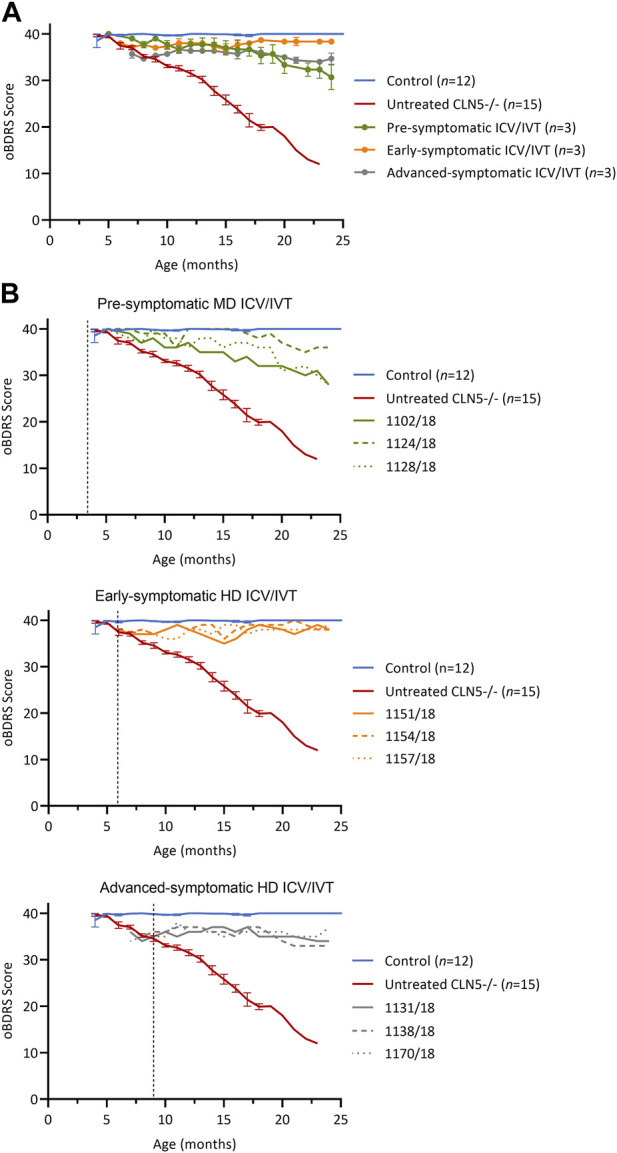
ICV/IVT scAAV9/oCLN5 slows or halts clinical progression. **(A)** Average oBDRS changes (±SEM) in ICV/IVT treated CLN5^−/−^ sheep were compared to average data from historical and concurrent healthy control CLN5^+/−^ (blue) and untreated CLN5^−/−^ sheep (red). Disease progression was halted for sheep treated at symptomatic disease stages with high ICV doses, whilst the lower ICV dose slowed clinical decline in pre-symptomatically treated sheep. **(B)** Individual oBDRS scores are displayed by treatment group (pre-symptomatic, green; early symptomatic, orange; advanced symptomatic, grey) at moderate (MD) or high (HD) doses. Dashed lines indicate treatment age.

The ovine Batten disease rating scale (oBDRS) (Mitchell et al., 2018) was used to clinically assess treated sheep (Figure 1). Age-matched untreated CLN5^−/−^ historical and concurrent controls demonstrated the natural progressive disease course and were euthanized at 18.7 ± 1.0 months of age, with oBDRS scores of 10–15. Age-matched healthy control CLN5^+/−^ sheep maintained oBDRS scores of 40 for the duration of the study.

Prior to treatment, pre-symptomatic sheep were phenotypically normal. Following treatment, they had an initial small decline in average oBDRS score, attributed to a progressive reduction in menace response, indicative of vision loss, in the untreated eye (Figure 1B, green). Sheep 1124/18 remained highly alert at 24 months of age and the only clinical deficit was a reduced menace response, resulting in difficulty negotiating stairs. The other two sheep in this cohort (1102/18 and 1128/18) lost their menace response in both eyes by 21–22 months, startle response by 22 months, and were unaware of human approach to 5 m by 24 months. Although these sheep had mild signs of reduced mentation and capability (e.g., confusion, somnolence, and reduced herding) and struggled to negotiate stairs, they did not have any further signs of manifest clinical disease.

Prior to ICV/IVT injection of scAAV9/oCLN5, the early symptomatic sheep had a mild clinical phenotype, including a slight head tilt and/or low head carriage, reduced menace responses, wariness of shadows, and crouching when travelling through a confined gateway; all indicative of the onset of vision loss. Following treatment, these animals remained clinically stable until sacrifice (Figure 1B, orange) with an average oBDRS score of 38 at 24 months of age (18 months post treatment). Deviations from normal were attributed to a loss of vision in the untreated eye. Otherwise, these sheep were highly alert, responsive to stimuli (loud noises, approaching humans and food rewards), and highly interactive with their cohorts.

Advanced symptomatic sheep had an overt clinical phenotype prior to treatment, displaying low head carriage and difficulty negotiating stairs. Visual deficits were apparent, and their herding instinct was diminished. Following treatment, the average oBDRS score for this cohort remained stable, with a pretreatment score of 35 and a score of 34 at 24 months old (15 months post treatment) (Figure 1B, grey). These sheep showed no further advancement in disease symptoms from enrolment.

### 3.4 Visual function and electroretinography

Analysis of the visual domain of the oBDRS showed that treatment with high-dose ICV/IVT scAAV9/oCLN5 sustained visual function in both the early and advanced symptomatic sheep compared with age-matched untreated CLN5^−/−^ sheep (Supplementary Figure S2). Efficacy was reduced for pre-symptomatic sheep that received an equivalent IVT dose but a lower ICV dose. Pre-symptomatic sheep 1102/18 and 1128/18 were classified as functionally blind (visual score ≤1) at 24 months of age and 1128/18 was actively running into objects (visual score 0).

Maze testing corroborated these findings (Supplementary Figure S3). After a period of habituation to learn the maze task, early symptomatic treated sheep demonstrated sustained cognitive and visual function, completing the maze in an average time of 28.8 ± 4.9 s over their lifetime, which was comparable to that of healthy CLN5^+/−^ control sheep (27.0 ± 3.1 s). Similarly, two out of the three advanced symptomatic treated sheep also completed the maze in an average of 27.3 ± 2.3 s over their lifetime. Times for the third animal in this cohort (1138/18) only slowed in the final two tests at 22 and 24 months of age, when this sheep failed to traverse one and two runs out of five respectively. In contrast, untreated CLN5^−/−^ sheep began to fail the maze from 19 months of age and could no longer participate from 21 months of age due to akinesia or confusion.

The pre-symptomatic sheep who had received an identical IVT dose but a ten-fold lower ICV dose than the other treatment cohorts demonstrated the lowest treatment effect. Only one pre-symptomatic treated sheep (1124/18) completed the maze with an average time of 28.3 ± 4.5 s over her lifetime. As indicated above, the other two sheep in this cohort lost vision in one or both eyes and failed to complete the maze fully from 22 to 24 months of age.

**FIGURE 2:**
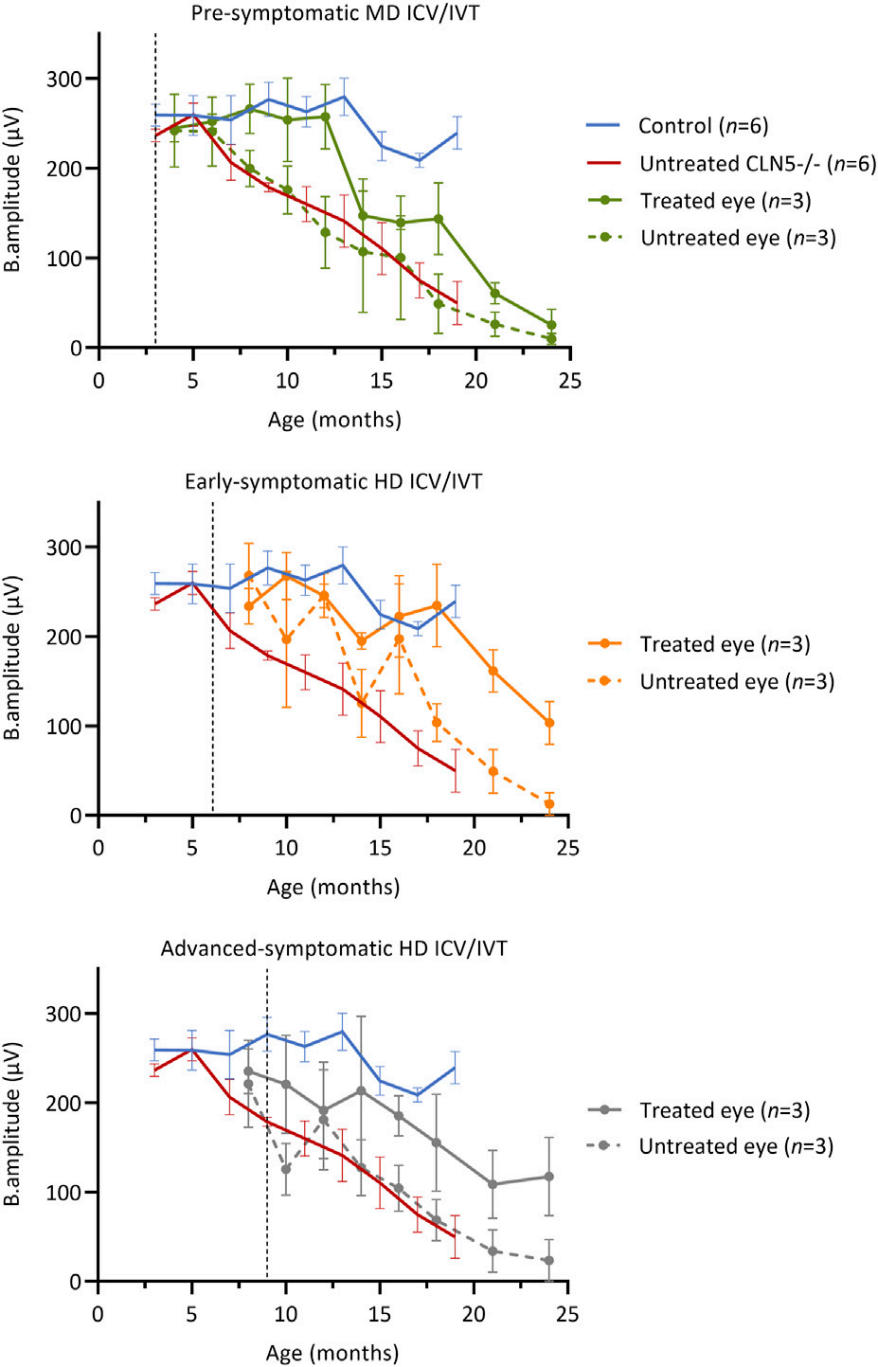
ICV/IVT scAAV9/oCLN5 slows retinal dysfunction. All sheep received an identical IVT dose into one eye, however ERG function was not maintained in pre-symptomatic treated sheep. The ten-fold higher co-administered ICV dose in early symptomatic and advanced symptomatic treated sheep afforded greater protection and slowed retinal dysfunction when average ERG b-wave amplitudes (±SEM) in the IVT scAAV9/oCLN5 treated (solid line) and untreated (dashed line) eyes of CLN5^−/−^ sheep were compared to average historic ERG data from healthy control CLN5^+/−^ (blue) and untreated CLN5^−/−^ sheep (red). Dashed lines indicate treatment age.

Longitudinal ERG (Russell et al., 2021) was also performed to monitor retinal physiology and function (Figure 2). IVT delivery of scAAV9/oCLN5 slowed stereotypical retinal dysfunction in both the early symptomatic and advanced symptomatic treated sheep. Their untreated eyes declined over time in line with data from untreated CLN5^−/−^ animals and, although there was a general trend towards loss of b-wave amplitude over time, the average rate of decline in the treated eye was almost half that of the untreated eye (Supplementary Table S10).

Despite receiving an identical IVT dose, but ten-fold lower ICV dose, retinal dysfunction was not halted in pre-symptomatic treated sheep. The average ERG amplitude from their treated eye initially tracked similarly to those of healthy control CLN5^+/−^ sheep, but over time declined steadily, and by 24 months of age it was similar to the untreated eye and close to or extinguished in all three sheep in this cohort.

### 3.5 Intracranial volume

**FIGURE 3:**
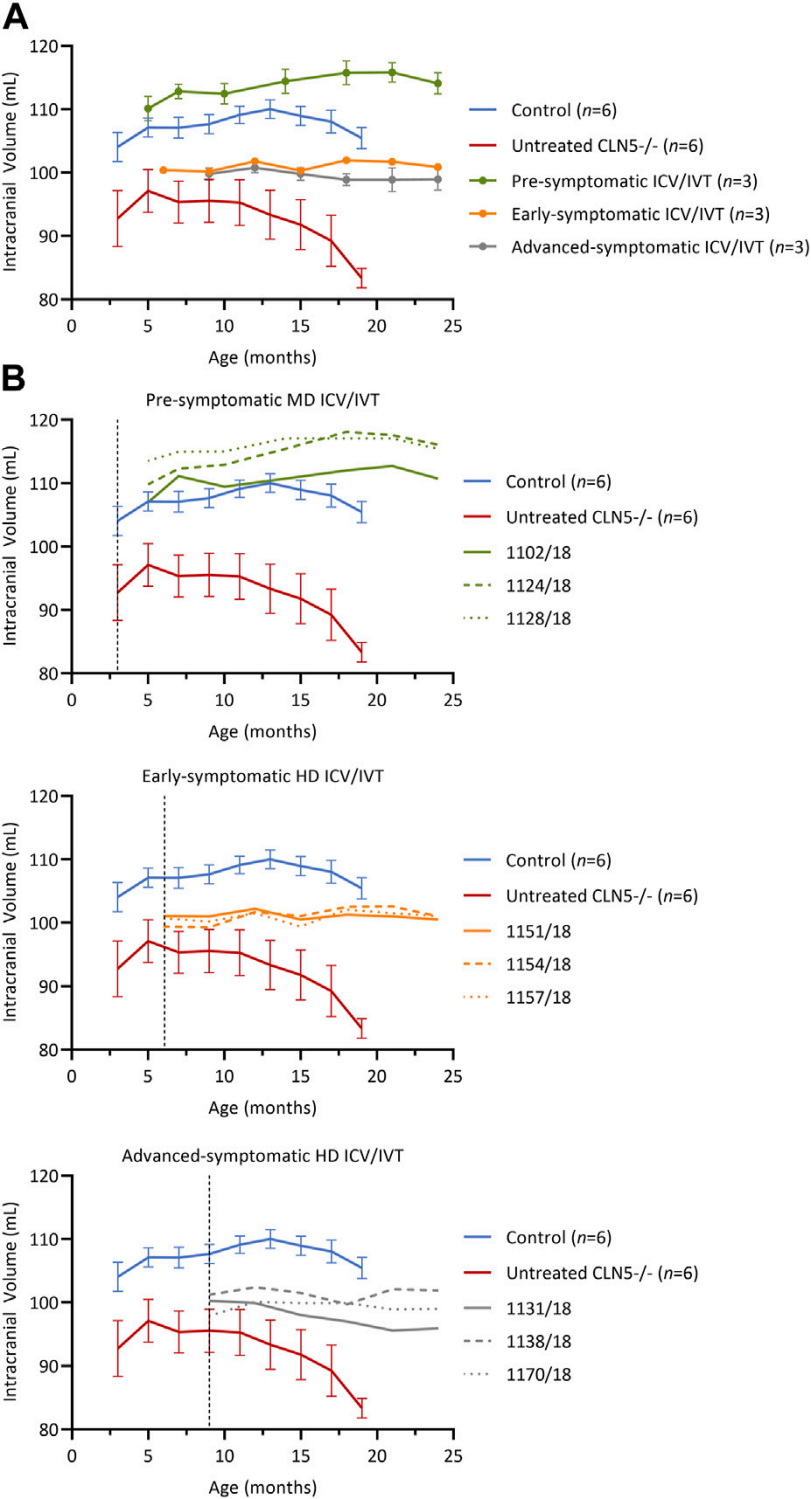
Intracranial volume was stabilized after ICV/IVT scAAV9/oCLN5. **(A)** Average intracranial volume changes (±SEM) in ICV/IVT treated CLN5^−/−^ sheep were stabilized when compared to average data from healthy control CLN5^+/−^ (blue) and untreated CLN5^−/−^ sheep (red). The greatest treatment effect was seen in animals treated pre-symptomatically, with volumes within the range of healthy control sheep. **(B)** Individual intracranial volumes are displayed by treatment group (pre-symptomatic, green; early symptomatic, orange; advanced symptomatic, grey) at moderate (MD) or high (HD) doses. Dashed lines indicate treatment age.

Longitudinal computed tomography (CT) scanning (Russell et al., 2018) was performed on all treated sheep and compared with historical data from healthy CLN5^+/−^ and untreated CLN5^−/−^ sheep (Figure 3).

Pre-symptomatic treated animals had normalised intracranial volumes at baseline (5 months) with a steady average gain of 3.9 ± 1.3 mL over their lifetime (Figure 3B, green). Early symptomatic treated sheep had stable intracranial volumes from baseline to 24 months, with volume changes ranging from −0.6 to +1.6 mL (Figure 3B, orange). Similarly, advanced symptomatic treated sheep maintained relatively stable brain volumes over their lifetime (Figure 3B, grey). One animal (1131/18) in the advanced symptomatic cohort displayed a slow decline in intracranial volume with a total loss of 4.4 mL from baseline to 24 months of age, but the other two animals in that cohort (1138/18 and 1170/18) had mild gains of 0.7 mL and 1.1 mL respectively.

Overall, the volume increases, or stabilisation observed in the majority of the ICV/IVT-treated sheep contrasted the rapid atrophy seen in untreated CLN5^−/−^ sheep, which causes an average loss of 9.4 mL of intracranial volume between 3 and 19 months of age.

### 3.6 Brain weight

All ICV/IVT treated sheep were euthanized at 24 months of age for neuropathological analyses. Fresh brain weights of the ICV/IVT treated sheep were compared with average historical data collected from age-matched healthy control CLN5^+/−^ and untreated CLN5^−/−^ sheep (Table 1). Whilst treated brains were smaller than healthy CLN5^+/−^ controls mediolaterally and had some mild gyral flattening, there was no evidence of gross lesions (Supplementary Figure S4). They weighed between 73% and 89% that of mean healthy control CLN5^+/−^ brain weight and were 16–33 g heavier than the mean age-matched untreated CLN5^−/−^ sheep brain weight.

**TABLE 1:** Summary of in-life and neuropathological efficacy endpoints following ICV/IVT scAAV9/oCLN5.

Study	Treatment	Sheep	Tx age (m)	End point (m)	Clinical description	Rate of decline^a^	Vision	Vision	Intracranial volume change (mL)^c^	Terminal brain	Cortical thickness^d^	Neuroinflammation	Lysosomal storage	CLN5 expression	Retinal pathology
							Left IVT^b^	Right^b^		Weight (g)					
Pre-symptomatic	MD ICV/IVT scAAV9/oCLN5	1102/18	3.6	24.3	Mild decline	−0.6	No	No	3.7	82.2	69%–73%	Low level	Significant reduction, ≤50% of untreated CLN5^−/−^	Mild to moderate across cortex, sparse in remaining CNS	Like untreated CLN5^−/−^
		1124/18	3.3	24.0	Stable	−0.2	Reduced	Reduced	6.3	95.3	77%–81%				Normal treated eye
		1128/18	3.3	24.0	Mild decline	−0.6	No	No	1.9	85.3	66%–80%				Like untreated CLN5^−/−^
Early symptomatic	HD ICV/IVT scAAV9/oCLN5	1151/18	6.3	24.0	Stable	0.0	Yes	No	−.06	85.1	73%–80%	Low level	Significant reduction, ≤50% of untreated CLN5^−/−^	High to extensive across cortex, mild in cerebellum and spinal cord, sparse in subcortex	Thicker ONL and less storage in treated eye
		1154/18	6.3	24.0	Stable	0.0	Yes	No	1.6	85.7	82%–89%				
		1157/18	6.1	23.8	Stable	0.1	Yes	Reduced	0.4	87.1	81%–92%				
Advanced symptomatic	HD ICV/IVT scAAV9/oCLN5	1131/18	9.5	24.1	Mild decline	0.0	No	No	−4.4	80.2	64%–67%	Low level	Significant reduction, ≥50% of untreated CLN5^−/−^	High to extensive across cortex, mild in cerebellum and spinal cord, sparse in subcortex	Like untreated CLN5^−/−^
		1138/18	9.4	24.0	Stable	0.0	Yes	Reduced	0.7	79.5	57%–72%				Thicker ONL and less storage in treated eye
		1170/18	8.9	23.5	Stable	0.1	Yes	Reduced	1.1	77.8	51%–72%				
Healthy control CLN5^+/−^ (n = 12)	Nil	N/A	N/A	>24	Normal	0.0	Vision in both eyes		3.3	106.1	100%	None	No present	Endogenous throughout CNS	Normal
										±1.5					
Untreated CLN5^−/−^ (n = 15)	Nil	N/A	N/A	18.6	Deceased, advanced disease	−1.7	No vision in either eye		−9.4	62.0	37%–42%	Extensive	Extensive	Absent	Thin, loss of ONL cells and extensive storage
										±1.0					

^a^ Rate of decline in oBDRS, units per month. For treated sheep this is from baseline (treatment age) to end point; for controls this is from 3 months to end point.

^b^ Treated sheep received an IVT, injection into their left eye, whilst their right eye was untreated.

^c^ Intracranial volume change from baseline (treatment age) to end point for treated sheep, or from 3 to 19 months of age for controls (Russell et al., 2018).

^d^ % cortical thickness of 24-month-old healthy control CLN5^+/−^

Abbreviations: CNS, central nervous system; HD, high dose; ICV, intracerebroventricular; m, months; MD, moderate dose; N/A, not applicable; oBDRS, ovine Batten disease rating scale; ONL, outer nuclear layer; Tx Treatment.

### 3.7 CLN5 biodistribution

The biodistribution of CLN5 expression was analysed in the central nervous system of ICV/IVT treated animals by immunostaining with sheep CLN5 antibodies (Supplementary Figure S5). No CLN5 protein expression was seen in the untreated CLN5^−/−^ sheep brain tissue whereas endogenous CLN5 protein was widely expressed throughout the healthy control CLN5^+/−^ brain, particularly in the cortical neurons, pyramidal cells of the hippocampus and cerebellar Purkinje cells. Punctate vector-driven CLN5 expression was observed in the treated brains, throughout the cortex, cerebellum and hippocampus, and predominantly in morphologically neuronal cells. Strong transduction was also evident in the motor neurons in the ventral horn of the entire spinal cord length.

**FIGURE 4:**
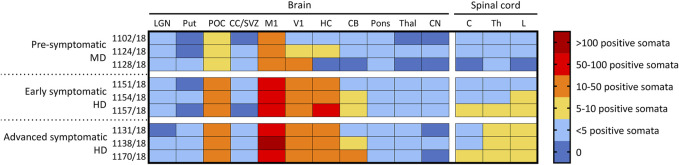
Ovine CLN5 protein is widely expressed in the brains of scAAV9/oCLN5 treated sheep. The number of CLN5-positive somata and fibres were quantified (zero to >100 CLN5-positive somata/0.57 mm^2^ field) in 11 brain and three spinal cord regions for 24-month-old CLN5^−/−^ sheep who were treated intracerebroventricularly and intravitreally with scAAV9/oCLN5 at 3 (pre-symptomatic), 6 (early symptomatic), or 9 (advanced symptomatic) months of age. A similar pattern of transduction was observed across the ICV/IVT treated brains, with greatest numbers of CLN5-positive cells in the cortical regions, but fewer transduced cells were seen in the pre-symptomatic treated brains which received ten-fold lower ICV doses. LGN, lateral geniculate nucleus; Put, putamen; POC, parieto-occipital cortex; CC/SVZ, corpus callosum/subventricular zone; M1, primary motor cortex; V1, primary visual cortex; HC, hippocampus; CB, cerebellum; Thal, thalamus; CN, caudate nucleus; C, cervical spinal cord; Th, thoracic spinal cord; L, lumbar spinal cord.

The number of CLN5-positive somata and fibres were semi-quantified for 11 distinct brain regions within the treated sheep brain hemispheres and three spinal cord levels (cervical, thoracic and lumbar) (Figure 4). Moderate to extensive CLN5 protein expression was observed in the motor, parieto-occipital and visual cortices and hippocampus of symptomatic sheep treated with scAAV9/oCLN5 at 6 or 9 months of age. Purkinje cells were the dominant population expressing CLN5 in the cerebellum whilst large subcortical structures, such as the thalamus, putamen or caudate nucleus had minimal expression. CLN5 expression was greater in the thoracic and lumbar regions than the cervical spinal cord.

A similar pattern of transduction was observed for the ICV/IVT sheep treated at a pre-symptomatic age (3 months) but with five to ten-fold less transduction efficiency (Figure 4). This correlated well with the fact that these animals received a ten-fold less ICV dose than the early and advanced symptomatic treated sheep.

### 3.8 Cortical thickness

**FIGURE 5:**
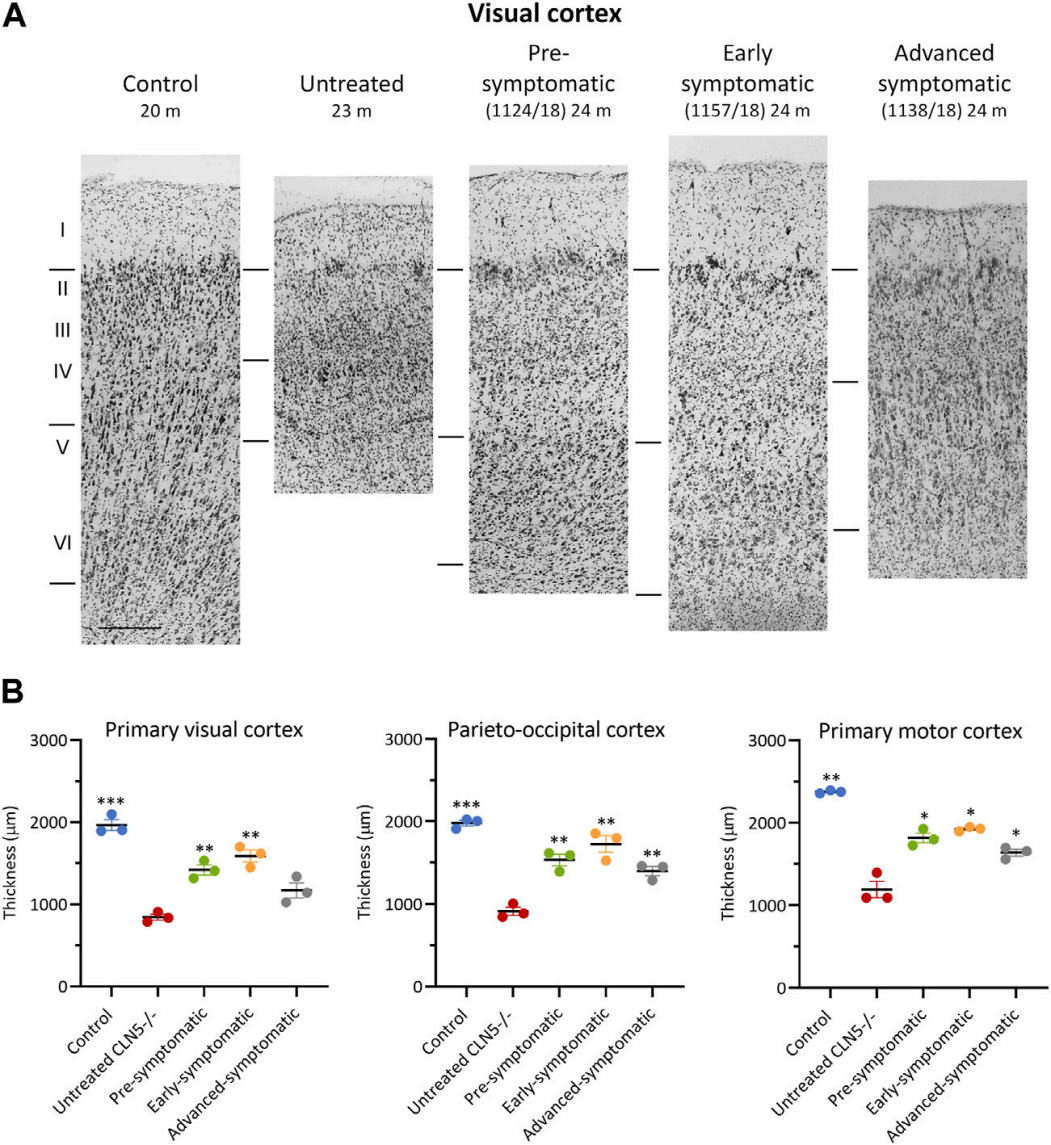
ICV/IVT scAAV9/oCLN5 attenuates cortical thinning. **(A)** Attenuation of cortical thinning can be seen in representative Nissl-stained images of the visual cortex of 24-month-old CLN5^−/−^ sheep who were treated intracerebroventricularly and intravitreally with scAAV9/oCLN5 at 3 (pre-symptomatic, green), 6 (early symptomatic, orange), or 9 (advanced symptomatic, grey) months of age compared to healthy control CLN5^+/−^ and untreated CLN5^−/−^ sheep. The top line marks the layer I/II boundary, middle line indicates the layer IV/V boundary, and lower line denotes the layer VI/white matter boundary. Scale bar (at bottom of 20 m control image) represents 200 µm. **(B)** Quantification of cortical thickness in three key regions which differentially undergo neurodegeneration in ovine CLN5 disease shows the treatment effect for individual animals in each treatment group. Significant differences to untreated CLN5^−/−^ are denoted by asterisks (***p < 0.001, **p < 0.01, *p < 0.05).

Cortical thicknesses in the ICV/IVT-treated CLN5^−/−^ sheep were compared with age-matched healthy control CLN5^+/−^ and untreated CLN5^−/−^ sheep (representative images are shown in Figure 5A). Marked atrophy and neuronal loss was evident in all regions of the untreated CLN5^−/−^ cerebral cortex. In contrast, a distinct laminar distribution of cells was observed across the cortical mantle of all ICV/IVT scAAV9/oCLN5-treated brains. Treated sheep cortical thicknesses were reduced compared with those of the healthy control CLN5^+/−^ brain, ranging from 69% to 87% of normal thickness in the motor and parieto-occipital cortices and 60%–81% in the visual cortex (Figure 5B). However, all values were significantly greater than those of an age-matched untreated CLN5^−/−^ animal. The greatest treatment effect on cortical thickness was consistently seen in the early symptomatic animals treated at 6 months of age.

**FIGURE 6:**
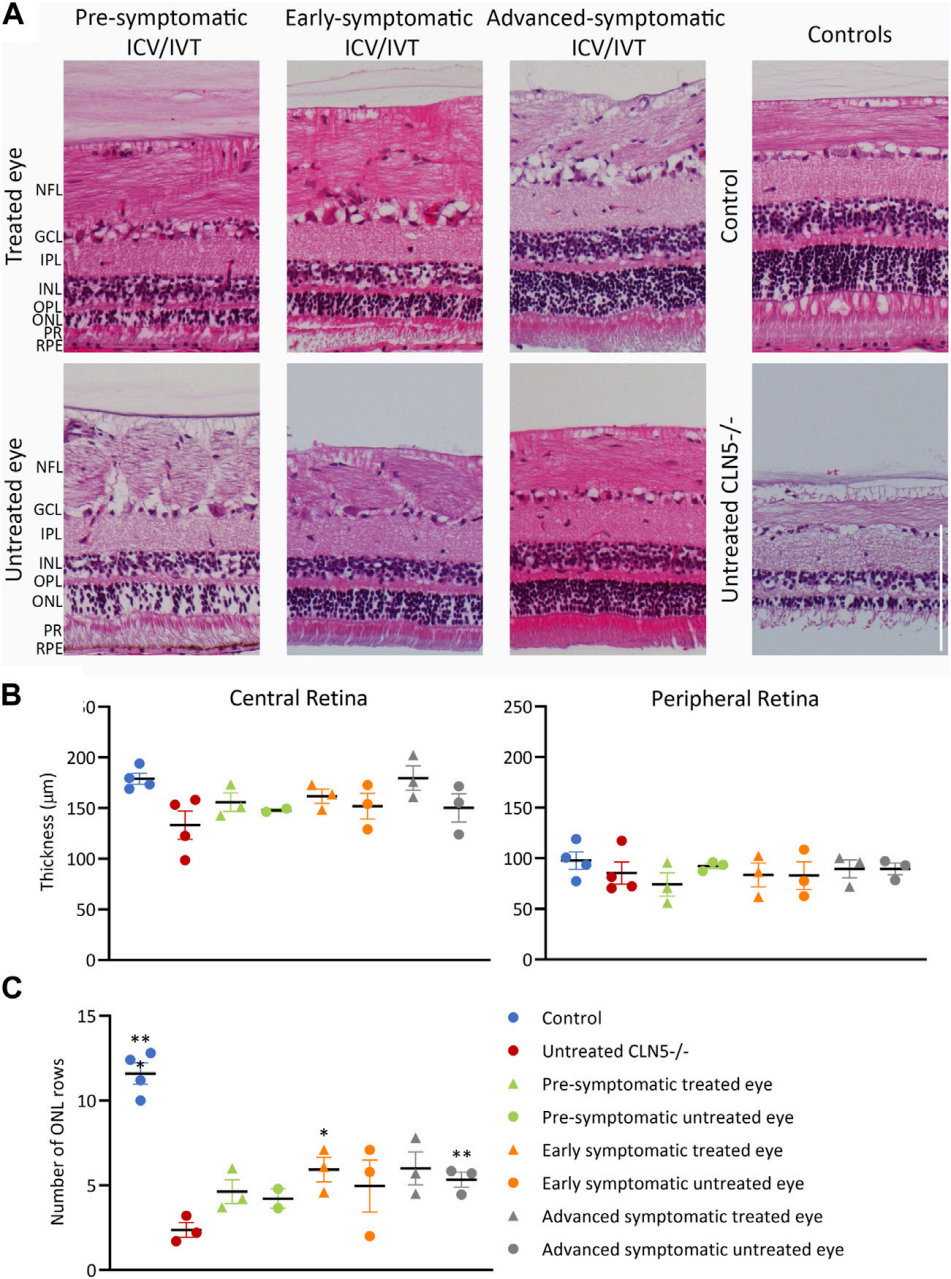
ICV/IVT scAAV9/oCLN5 did not have a significant effect on retinal pathology. **(A)** Representative hematoxylin and eosin-stained central retinal sections from the treated and untreated eye of 24-month-old ICV/IVT treated CLN5^−/−^ sheep were compared to age-matched healthy control CLN5^+/−^ and untreated CLN5^−/−^ retina. NFL, nerve fiber layer; GCL, ganglion cell layer; IPL, inner plexiform layer; INL, inner nuclear layer; OPL, outer plexiform layer; ONL, outer nuclear layer; PR, photoreceptor layer; RPE, retinal pigment epithelium. Scale bar (in lower right image) represents 100 µm. **(B)** Quantification of retinal thickness (µm) in the central and peripheral retina did not show any significant difference between treated and untreated eyes. **(C)**. Quantification of photoreceptor nuclei in the ONL of the retina of CLN5^−/−^ sheep who were treated at pre-symptomatic, early symptomatic, or advanced symptomatic disease stages also showed no difference between treated and untreated eyes. Significant differences compared to untreated CLN5^−/−^ sheep are denoted by asterisks (***p < 0.001, **p < 0.01).

Despite the phenotypic differences observed between treated and untreated eyes, there was no significant difference in central retinal thickness when the treated eyes from the ICV/IVT sheep were compared with their untreated eyes (Figure 6). There was a trend towards a thicker retina in the treated eyes, with the largest difference between the two eyes seen in the advanced symptomatic cohort. Two sheep in this cohort (1138/18 and 1170/18) had a greater retinal thickness in their treated eyes compared with their untreated eyes (Figure 6B); while sheep 1131/18 had a thinner treated central retina, which corroborates with clinical loss of vision in this animal.

Results from the pre-symptomatic group were confounded by the lack of a central retina sample from the untreated right eye of sheep 1102/18, which was lost during processing for histology. This animal had a near healthy retinal thickness in the treated eye. There was no statistical difference in retinal thickness between eyes for the best (1124/18) and worst (1128/18) clinically performing sheep in this cohort.

In human and ovine NCL, retinal atrophy results from an almost complete loss of rod and cone photoreceptors (Weleber et al., 2004; Murray and Mitchell, 2022) hence photoreceptor nuclei were counted in the outer nuclear layer (ONL) of the sheep central retina. Counts showed that the number of nuclei was generally higher in both eyes of treated animals, with average counts of 4.6, 5.9 and 6.0 for the pre-, early and advanced symptomatic ICV/IVT treated animals respectively, compared with an average count of 2.4 ONL rows in the age-matched untreated CLN5^−/−^ eyes (Figure 6C).

### 3.9 Neuroinflammation

The neuroinflammatory response in the ICV/IVT treated sheep was compared with age-matched healthy control CLN5^+/−^ and untreated CLN5^−/−^ sheep using an astrocytic marker (GFAP) and a microglial marker (GSB4) (Supplementary Figures S6, S7). Quantitative threshold analyses confirmed that ICV/IVT scAAV9/oCLN5 gene therapy suppressed neuroinflammation (Supplementary Figures S6, S7). Specifically, GFAP staining revealed a dense glial meshwork of hypertrophic activated astrocytes across the upper cortical layers of the untreated CLN5^−/−^ brain, which was not present in the scAAV9/oCLN5-treated brains (Supplementary Figure S6). Astroglial morphologies in the brains of treated sheep more closely resembled those seen in the control CLN5^+/−^ brain, being fibrous in the white matter and highly branched and protoplasmic with numerous processes in the cortical grey matter. Some hypertrophy and clusters of activated glia were detected in cortical layers II-III of treated sheep. However, the total GFAP immunoreactivity across the grey matter layers of the visual, parieto-occipital, and motor cortices of ICV/IVT treated animals was only half that seen in untreated CLN5^−/−^ animals of a similar age, indicating significant amelioration in disease-associated astrocytic activation.

A similar trend was seen with another neuroinflammatory marker, GSB4. A distinct reduction in activated microglia was observed in the cortical parenchyma of treated animals and this trend reached significance in the parieto-occipital and motor cortices (Supplementary Figure S7). There was no evidence of the clusters of GSB4-positive activated amoeboid microglia and hypertrophied brain macrophages that are seen in the untreated CLN5^−/−^ brain as two conspicuous bands within cortical layers II-III and V-VI (Supplementary Figure S7A).

### 3.10 Lysosomal storage

**FIGURE 7:**
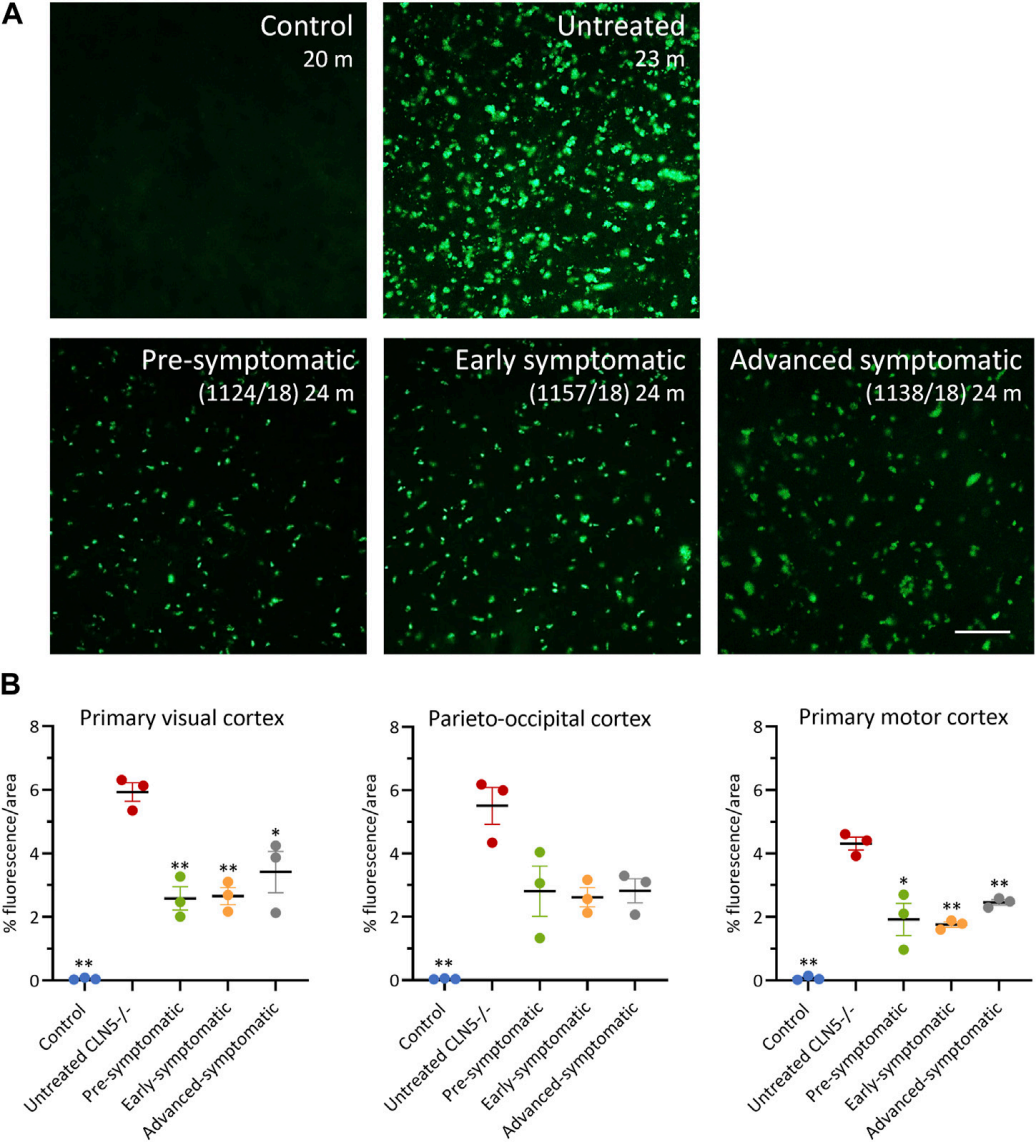
ICV/IVT scAAV9/oCLN5 attenuates lysosomal storage burden. **(A)** Levels of lysosomal storage material were reduced in representative cerebral cortex sections of 24-month-old CLN5^−/−^ sheep treated intracerebroventricularly and intravitreally with scAAV9/oCLN5 at 3 (pre-symptomatic, green), 6 (early symptomatic, orange), or 9 (advanced symptomatic, grey) months of age when compared to untreated CLN5^−/−^ sheep brain. Scale bar (in right-most image) represents 50 µm. **(B)** Quantification of the mean percentage area of fluorescence in three key brain regions shows the treatment effect for individual animals in each group. Vertical bars denote ±SEM. Significant differences to untreated CLN5^−/−^ are denoted by asterisks (***p < 0.001, **p < 0.01, *p < 0.05).

Lysosomal storage was absent in healthy control CLN5^+/−^ sheep brain yet punctate, globular storage bodies were densely packed into the cortical neurons and glial-like cells of the untreated CLN5^−/−^ sheep brain, as well as in the perikarya of cerebellar Purkinje cells. The ICV/IVT treatment significantly reduced this storage body accumulation in the primary visual and motor cortices (Figure 7). This was particularly apparent in the pre-symptomatic and early symptomatic treated cohorts, whose cortices had half the lysosomal burden seen in age-matched untreated CLN5^−/−^ sheep.

Thresholding analysis was also performed on the treated and untreated retina from the ICV/IVT treated sheep (Supplementary Figure S8). In retina with fluorescent burden, distinct puncta were predominantly observed in the retinal ganglion cells; more diffuse storage accumulation was seen in the inner nuclear, outer nuclear, and plexiform layers. The retinal pigment epithelium (RPE) and inner segment/outer segment photoreceptor (IS/OS) layers were excluded from the analyses because high levels of endogenous fluorescence present in these layers in the healthy control CLN5^+/−^ sheep eye precluded a comparison.

Retinal fluorescence in both eyes of pre-symptomatic ICV/IVT treated sheep 1102/18 and 1128/18 was similar to that in untreated CLN5^−/−^ sheep (1.2%–2.2% vs. 1.30%, respectively: Supplementary Figure S8). In contrast, sheep 1124/18 had much less storage in the treated retina and more closely resembled a healthy control CLN5^+/−^ eye (0.22% vs. 0.16%, respectfully). Storage accumulation was lower in the retina of the treated eye compared with the untreated eye for all early symptomatic treated sheep although this did not reach statistical significance (Supplementary Figure S8B). Surprisingly, both eyes of the advanced symptomatic treated animals also had much lower levels of fluorescence compared with age-matched untreated CLN5^−/−^ sheep (Supplementary Figure S8B). While there was a trend for less storage body accumulation in the treated retina compared with the untreated retina for the advanced symptomatic sheep, statistical significance was not reached.

## 4 Discussion

This study was the first to evaluate combined ICV/IVT administration of a gene therapy for CLN5 disease in a large animal model representing pre-symptomatic as well as early and advanced symptomatic stages. To date, nine CLN5^−/−^ sheep have received the dual treatment with scAAV9/oCLN5, and a summary of the results is presented in Table 1. The dual treatment slowed disease progression, attenuated CLN5 disease pathology, and preserved vision. These data, combined with previous pre-clinical ICV studies in CLN5^−/−^ sheep (Mitchell et al., 2018; Mitchell et al., 2023a), contributed to the clearance of the *CLN5* gene therapy product as an investigational new drug (IND) for the treatment of both the neurological and retinal components of human CLN5 Batten disease and supported the initiation of the current Phase I/II *CLN5* gene therapy clinical trial testing scAAV9/hCLN5 dual route ICV/IVT administration (Clinicaltrials.gov identifier: NCT05228145).

The present study employed a naturally occurring sheep model of CLN5 Batten disease. Although a CLN5 exon 3 knock-out mouse model (Kopra et al., 2004) has provided insights into the spatiotemporal expression of CLN5 in the central nervous system (CNS) (Holmberg et al., 2004), these mice lack the severe brain atrophy characteristic of the sheep and human diseases. In contrast, CLN5^−/−^ sheep share the main neuropathological features of the human disease, including progressive brain atrophy and loss of vision at approximately 11 months of age (Jolly et al., 2002; Frugier et al., 2008). In addition, sheep are similar in size to humans, weighing 3.5–4.5 kg at birth and growing to 80–110 kg in adulthood, with comparable spine dimensions, cerebrospinal fluid volumes, and pulmonary and cardiac parameters (Wilke et al., 1997; Scheerlinck et al., 2008; Wilson et al., 2017). Furthermore, the gyrencephalic ovine brain is similar in physical organization to the human brain and in size to non-human primate brains, providing a good approximation for dose requirements, vector delivery, and distribution to inform clinical translation.

Previous studies have shown that delivery of *CLN5* gene therapy to the CLN5^−/−^ sheep brain via ICV administration alone is sufficient to halt or slow disease onset and progression, intracranial volume loss, and ameliorate disease pathology (Mitchell et al., 2018; Mitchell et al., 2023a). The results of this study indicate a dose-dependent transduction efficiency and correlated improved efficacy with the high ICV dose in the early and advanced symptomatic sheep as compared with the moderate dose evaluated in the pre-symptomatic animals. Pre-symptomatic sheep received a ten-fold lower ICV dose than the other treatment groups, which normalized their intracranial volumes but resulted in the greatest rate of clinical decline from injection (−0.5 oBDRS points per month compared with no average change in the older symptomatic treated cohorts). Immunohistochemical analyses of CNS tissues demonstrated far fewer CLN5-positive cells in the pre-symptomatic treated cohort, reflecting their lesser ICV dose. Previous attempts at a moderate dose ICV delivery (2.4 × 10^11^ vg) in 9-month-old CLN5^−/−^ sheep with advanced disease symptoms resulted in only one animal responding favorably (Mitchell et al., 2023a). In the current study, however, a higher dose was administered to symptomatic sheep, and all three of the early symptomatic treated sheep and two of the three advanced symptomatic treated sheep were clinically stable at 24 months of age. The third advanced symptomatic sheep presented with only mild decline. In addition, the brains of sheep treated at early and advanced symptomatic stages had extensive cortical CLN5 transduction, only mild neuroinflammation and significantly reduced lysosomal storage compared with age-matched untreated CLN5^−/−^ sheep. Regions like the primary motor, visual and parieto-occipital cortices and hippocampus were particularly well-targeted by the ICV delivery, and at the higher dose, CLN5-positive cells were evident along the full length of the spinal cord. The relative success of this higher dose ICV treatment in both early and advanced symptomatic sheep is extremely promising for its translation to symptomatic CLN5 patients.

Although all nine sheep received the same IVT dose, greater attenuation of retinal pathology was observed in sheep treated at the later disease stages (6 and 9 months) with a higher ICV dose compared with those treated at the pre-symptomatic stage with a lower ICV dose. Five out of six sheep in the symptomatic cohorts had thicker total retina and outer nuclear layers in the treated eyes; however, retinal thinning and lysosomal storage was attenuated in both eyes (treated and untreated) compared with untreated CLN5^−/−^ sheep. One possible mechanism is that retrograde axonal transport of the vector from the brain to the eye, via the optic nerve, is occurring and this might explain why the animals with the higher ICV brain doses showed the greatest functional and pathological correction in the retina. CLN5-positive transduced cells were found throughout key visual pathway structures in the brain, including the lateral geniculate nucleus (LGN) and primary visual cortex, therefore it is possible that this retrograde transport occurs between the LGN, where ganglion cells terminate, and the retina, where ganglion cell bodies are located.

Natural history electroretinography studies in untreated CLN5^−/−^ sheep showed that retinal dysfunction begins around 5 months of age (Russell et al., 2021), and this dysfunction is accompanied by progressive loss of vulnerable neurons in the primary visual cortex and outer nuclear layer and photoreceptor cells in the retina (Murray and Mitchell, 2022; Mitchell et al., 2023b). IVT administration alone was not sufficient to prevent vision loss in CLN5^−/−^ sheep, likely due to cortical blindness since the CNS remained untreated (Murray et al., 2021). However, both moderate- and high-dose ICV delivery of *CLN5* gene therapy likely prevented the cortical neurodegenerative-driven blindness seen in untreated CLN5^−/−^ sheep as all treated animals had improved visual function compared with untreated controls, confirming the importance of treating the CNS in combination with the eye to prevent loss of visual function. Moreover, IVT delivery may have impacted different aspects of retinal pathology, such as retinal thinning and lysosomal storage, compared to ICV administration.

Immunohistochemical localisation of CLN5 protein in the sheep retina has not been possible to date. Currently available antibodies against ovine CLN5 work in central nervous system tissues but do not produce staining in the retina, making it difficult to assess number and types of cells transduced in the current study. In NCLs, even minor increases in functional protein levels have been shown to be effective in correcting phenotype (Parenti et al., 2015); therefore, the IVT delivery route may transduce the inner retinal cells layers, such as the ganglion cell layer, and the transduced cells secrete sufficient CLN5 protein to protect outer retinal cells, including the photoreceptors, which are severely compromised in CLN5 patients.

The first of two IND-enabling studies in CLN5^−/−^ sheep demonstrated the long-term safety and efficacy of ICV scAAV9/oCLN5 gene therapy when delivered at pre-symptomatic, early symptomatic, and more advanced symptomatic disease stages (Mitchell et al., 2023a). Here, the second study demonstrated proof-of-concept for the dual ICV/IVT delivery route. Disease progression and stereotypical brain atrophy were significantly slowed, and disease-associated pathology was greatly attenuated with the combined routes of gene therapy. Vision was preserved in the majority of treated sheep up to 24 months of age with improvements in retinal pathology and function. Doses used in sheep in the current study will be used to extrapolate optimal doses for the Phase I/II clinical trial, with brain volume selected as the most reliable dosing metric. Collectively, these findings indicate great translational promise for extending and improving vision and other core symptoms in both pre-symptomatic and symptomatic CLN5 patients.

## Data Availability

The raw data supporting the conclusion of this article will be made available by the authors, without undue reservation.

## References

[B1] Autti T. Raininko R. Launes J. Nuutila A. Santavuori P. (1992). Jansky-Bielschowsky variant disease: CT, MRI, and SPECT findings. Pediatr. Neurol. 8, 121–126. 10.1016/0887-8994(92)90032-T 1580955

[B2] Basak I. Wicky H. E. McDonald K. O. Xu J. B. Palmer J. E. Best H. L. (2021). A lysosomal enigma CLN5 and its significance in understanding neuronal ceroid lipofuscinosis. Cell. Mol. Life Sci. 78, 4735–4763. 10.1007/s00018-021-03813-x 33792748 PMC8195759

[B3] Bates D. Mächler M. Bolker B. Walker S. (2015). Finding patients before they crash: the next major opportunity to improve patient safety. J. Stat. Softw. 67, 1–3. 10.1136/bmjqs-2014-003499 25249637

[B4] Federov A. Beichel R. Kalpathy-Cramer J. Finet J. Fillion-Robin J.-C. Pujol S. (2012). 3D Slicer as an image computing platform for the quantitative imaging network. Magn. Reson. Imaging 30, 1323–1341. 10.1016/j.mri.2012.05.001 22770690 PMC3466397

[B5] Frugier T. Mitchell N. L. Tammen I. Houweling P. J. Arthur D. G. Kay G. W. (2008). A new large animal model of CLN5 neuronal ceroid lipofuscinosis in Borderdale sheep is caused by a nucleotide substitution at a consensus splice site (c.571+1G>A) leading to excision of exon 3. Neurobiol. Dis. 29, 306–315. 10.1016/j.nbd.2007.09.006 17988881 PMC2249613

[B6] Holmberg V. Jalanko A. Isosomppi J. Fabritius A. L. Peltonen L. Kopra O. (2004). The mouse ortholog of the neuronal ceroid lipofuscinosis CLN5 gene encodes a soluble lysosomal glycoprotein expressed in the developing brain. Neurobiol. Dis. 16, 29–40. 10.1016/j.nbd.2003.12.019 15207259

[B7] Holmberg V. Lauronen L. Autti T. Santavuori P. Savukoski M. Uvebrant P. (2000). Phenotype-genotype correlation in eight patients with Finnish variant late infantile NCL (CLN5). Neurology 55, 579–581. 10.1212/wnl.55.4.579 10953198

[B8] Jolly R. D. Arthur D. G. Kay G. W. Palmer D. N. (2002). Neuronal ceroid-lipofuscinosis in Borderdale sheep. N. Z. Vet. J. 50, 199–202. 10.1080/00480169.2002.36311 16032271

[B9] Kopra O. Vesa J. von Schantz C. Manninen T. Minye H. Fabritius A. L. (2004). A mouse model for Finnish variant late infantile neuronal ceroid lipofuscinosis, CLN5, reveals neuropathology associated with early aging. Hum. Mol. Genet. 13, 2893–2906. 10.1093/hmg/ddh312 15459177

[B10] Linterman K. S. Palmer D. N. Kay G. W. Barry L. A. Mitchell N. L. McFarlane R. G. (2011). Lentiviral-mediated gene transfer to the sheep brain: Implications for gene therapy in Batten disease. Hum. Gene Ther. 22, 1011–1020. 10.1089/hum.2011.026 21595499 PMC3159522

[B11] Mitchell N. L. Murray S. J. Wellby M. P. Barrell G. K. Russell K. N. Deane A. R. (2023a). Long-term safety and dose escalation of intracerebroventricular CLN5 gene therapy in sheep supports clinical translation for CLN5 Batten disease. Front. Genet. 14, 1212228. 10.3389/fgene.2023.1212228 37614821 PMC10442658

[B12] Mitchell N. L. Russell K. N. Barrell G. K. Tammen I. Palmer D. N. (2023b). Characterization of neuropathology in ovine CLN5 and CLN6 neuronal ceroid lipofuscinoses (Batten disease). Dev. Neurobiol. 83, 127–142. 10.1002/dneu.22918 37246363

[B13] Mitchell N. L. Russell K. N. Wellby M. P. Wicky H. E. Schoderboeck L. Barrell G. K. (2018). Longitudinal In Vivo Monitoring of the CNS Demonstrates the Efficacy of Gene Therapy in a Sheep Model of CLN5 Batten Disease. Mol. Ther. 26, 2366–2378. 10.1016/j.ymthe.2018.07.015 30078766 PMC6171082

[B14] Murray S. J. Mitchell N. L. (2022). Natural history of retinal degeneration in ovine models of CLN5 and CLN6 neuronal ceroid lipofuscinoses. Sci. Rep. 12, 3670–3713. 10.1038/s41598-022-07612-7 35256654 PMC8901734

[B15] Murray S. J. Russell K. N. Melzer T. R. Gray S. J. Heap S. J. Palmer D. N. (2021). Intravitreal gene therapy protects against retinal dysfunction and degeneration in sheep with CLN5 Batten disease. Exp. Eye Res. 207, 108600. 10.1016/j.exer.2021.108600 33930398

[B16] Oswald M. J. Palmer D. N. Kay G. W. Shemilt S. J. A. Rezaie P. Cooper J. D. (2005). Glial activation spreads from specific cerebral foci and precedes neurodegeneration in presymptomatic ovine neuronal ceroid lipofuscinosis (CLN6). Neurobiol. Dis. 20, 49–63. 10.1016/j.nbd.2005.01.025 16137566

[B17] Parenti G. Andria G. Ballabio A. (2015). Lysosomal Storage Diseases: From pathophysiology to therapy. Annu. Rev. Med. 66, 471–486. 10.1146/annurev-med-122313-085916 25587658

[B18] R Core Team (2021). R: A Language and Environment for Statistical Computing. Available at: https://www.R-project.org/.

[B19] Russell K. N. Mitchell N. L. Anderson N. G. Bunt C. R. Wellby M. P. Melzer T. R. (2018). Computed tomography provides enhanced techniques for longitudinal monitoring of progressive intracranial volume loss associated with regional neurodegeneration in ovine neuronal ceroid lipofuscinoses. Brain Behav. 8, e01096. 10.1002/brb3.1096 30136763 PMC6160654

[B20] Russell K. N. Mitchell N. L. Wellby M. P. Barrell G. K. Palmer D. N. (2021). Electroretinography data from ovine models of CLN5 and CLN6 neuronal ceroid lipofuscinoses. Data Br. 37, 107188. 10.1016/j.dib.2021.107188 PMC8187955 34141843

[B21] Sands M. S. Davidson B. L. (2006). Gene therapy for lysosomal storage diseases. Mol. Ther. 13, 839–849. 10.1016/j.ymthe.2006.01.006 16545619

[B22] Scheerlinck J.-P. Y. Snibson K. J. Bowles V. M. Sutton P. (2008). Biomedical applications of sheep models: from asthma to vaccines. Trends Biotechnol. 26, 259–266. 10.1016/j.tibtech.2008.02.002 18353472

[B23] Tyynelä J. Cooper J. D. Khan M. N. Shemilts S. J. A. Haltia M. (2004). Hippocampal pathology in the human neuronal ceroid-lipofuscinoses: distinct patterns of storage deposition, neurodegeneration and glial activation. Brain Pathol. 14, 349–357. 10.1111/j.1750-3639.2004.tb00077.x 15605981 PMC8095893

[B24] Tyynelä J. Suopanki J. Santavuori P. Baumann M. Haltia M. (1997). Variant late infantile neuronal ceroid-lipofuscinosis: pathology and biochemistry. J. Neuropathol. Exp. Neurol. 56, 369–375. 10.1097/00005072-199704000-00005 9100667

[B25] Weleber R. G. Gupta N. Trzupek K. M. Wepner M. S. Kurz D. E. Milam A. H. (2004). Electroretinographic and clinicopathologic correlations of retinal dysfunction in infantile neuronal ceroid lipofuscinosis (infantile Batten disease). Mol. Genet. Metab. 83, 128–137. 10.1016/j.ymgme.2004.06.019 15464427

[B26] Wilke H.-J. Kettler A. Wenger K. le C. (1997). Anatomy of the sheep spine and its comparison to the human spine. Anat. Rec. 247, 542–555. 10.1002/(SICI)1097-0185(199704)247:4<542:AID-AR13>3.0.CO;2-P 9096794

[B27] Wilson S. Abode-Iyamah K. O. Miller J. W. Reddy C. G. Safayi S. Fredericks D. C. (2017). An ovine model of spinal cord injury. J. Spinal Cord. Med. 40, 346–360. 10.1080/10790268.2016.1222475 27759502 PMC5472023

[B28] Xin W. Mullen T. E. Kiely R. Min J. Feng X. Cao Y. (2010). CLN5 mutations are frequent in juvenile and late-onset non-Finnish patients with NCL. Neurology 74, 565–571. 10.1212/WNL.0b013e3181cff70d 20157158

