# Supplementary material for: Efficacy of dual intracerebroventricular and intravitreal CLN5 gene therapy in sheep prompts the first clinical trial to treat CLN5 Batten disease
Source: Front Pharmacol. 2023 Oct 24;14:1212235. doi: 10.3389/fphar.2023.1212235 (PMC10628725; doi:10.3389/fphar.2023.1212235)
Supplement: Supplementary file 1 [file DataSheet1.PDF]

Supplementary Material

Efficacy of dual intracerebroventricular and intravitreal *CLN5* gene therapy in sheep supports the first clinical trial to treat CLN5 Batten disease.

Samantha J. Murray, Martin P. Wellby, Graham K. Barrell, Katharina N. Russell, Ashley R. Deane, John R. Wynyard, Steven J. Gray, David N. Palmer, Nadia L. Mitchell*

*** Correspondence:** Nadia Mitchell: Nadia.Mitchell@lincoln.ac.nz

Supplementary Table 1. Combination intracerebroventricular/intravitreal *CLN5* gene therapy study design.

Study	Vector and transgene	Treatment	Dose	Total ICV dose (vg)	ICV volume (µl)	Total IVT dose (vg)	IVT volume (µl)	Sheep n	Genetic status	Treatment age (months)	Viral titer (vg/mL)
Pre-symptomatic	scAAV9/oCLN5	Bilateral ICV/ Unilateral IVT	MD	2.9×10^{11}	800	6.5×10^{10}	100	3	$CLN5^{-/-}$	3.3-3.6	1.4×10^{13}
Early symptomatic	scAAV9/oCLN5	Bilateral ICV/ Unilateral IVT	HD	3.3×10^{12}	800	6.5×10^{10}	100	3	$CLN5^{-/-}$	6.0	1.4×10^{13}
Advanced symptomatic	scAAV9/oCLN5	Bilateral ICV/ Unilateral IVT	HD	3.3×10^{12}	800	6.5×10^{10}	100	3	$CLN5^{-/-}$	8.8-9.0	1.4×10^{13}
Control	N/A	Nil	N/A	N/A	N/A	N/A	N/A	3	$CLN5^{+/-}$	N/A	N/A
Affected	N/A	Nil	N/A	N/A	N/A	N/A	N/A	4	$CLN5^{-/-}$	N/A	N/A

Abbreviations: HD high dose; ICV intracerebroventricular; m months; MD moderate dose; n number; N/A not applicable; vg viral genomes

Supplementary Table 2. Visual assessment in CLN5$^{-/-}$ sheep.

**Visual trait**	**Grade**				
	**4**	**3**	**2**	**1**	**0**
Head tilt	Absent	Present			
Response to shadows	Absent	Wary			
Visual tracking	Present	Reduced	Lost		
Menace response	Present	Reduced or absent in 1 eye	Reduced or absent in both eyes		
Dazzle response	Present		Reduced		Absent
Corneal/palpebral reflex	Present		Reduced		Absent
Pupillary light reflex	Present			Reduced	Absent
Funduscopic changes	Absent			Present	
Electroretinography response	Intact			Obviously reduced	Absent
Hitting objects	Absent				Present

Supplementary Table 3. Cortical thickness following ICV/IVT treatment with scAAV9/oCLN5. Mean cortical thickness measurements (± SEM) for $CLN5^{-/-}$ sheep treated intracerebroventricularly and intravitreally with scAAV9/oCLN5 at 3 (pre-symptomatic), 6 (early symptomatic), or 9 (advanced-symptomatic) months of age were compared to healthy control $CLN5^{+/-}$ and untreated $CLN5^{-/-}$ sheep. At least 25 thickness measurements were taken per hemisphere per animal through three different cortical regions on Nissl-stained sections.

Treatment	Sheep	Age at death (m)	Visual cortex		Parieto-occipital cortex		Motor cortex	
			Thickness (µm)	SEM	Thickness (µm)	SEM	Thickness (µm)	SEM
Healthy control $CLN5^{+/-}$	39C	24.2	2097	50	2006	27	2394	83
	1103C	24.0	1893	67	2023	60	2356	40
	1111C	24.1	1940	44	1912	25	2376	53
Untreated $CLN5^{-/-}$	1104U	23.7	788	25	845	21	1095	16
	1109U	23.2	837	24	887	21	1395	63
	1116U	23.4	908	28	1007	20	1094	35
Pre-symptomatic	1102/18	24.3	1408	24	1395	24	1726	29
	1124/18	24.0	1532	29	1617	17	1925	28
	1128/18	24.0	1319	24	1591	17	1798	19
Early symptomatic	1151/18	24.0	1449	19	1527	25	1895	15
	1154/18	24.0	1617	29	1800	26	1948	27
	1157/18	23.8	1698	20	1855	19	1930	16
Advanced symptomatic	1131/18	24.1	1340	19	1288	17	1559	40
	1138/18	24.0	1144	27	1451	28	1697	18
	1170/18	23.5	1026	18	1458	24	1657	24

Abbreviations: m months

Supplementary Table 4. Retinal thickness following ICV/IVT treatment with scAAV9/oCLN5. Mean retinal thickness measurements (± SEM) for CLN5$^{-/-}$ sheep treated intracerebroventricularly and intravitreally with scAAV9/oCLN5 at 3 (pre-symptomatic), 6 (early symptomatic), or 9 (advanced-symptomatic) months of age were compared to healthy control CLN5$^{+/-}$ and untreated CLN5$^{-/-}$ sheep. At least 20 total thickness measurements were taken through the central and peripheral retina per animal on hematoxylin and eosin-stained sections.

Treatment	Sheep	Age at death (m)	Central		Peripheral	
			Thickness	SEM	Thickness	SEM
Healthy control CLN5$^{+/-}$	15-4C	24.0	194.0	6.9	100.7	0.7
	15-2C	24.0	179.8	6.2	77.4	0.9
	205-4C	24.0	169.1	6.9	119.0	1.7
	205-2C	24.0	173.5	5.3	93.7	0.8
Untreated CLN5$^{-/-}$	1114U	18.3	158.3	2.7	117.4	1.5
	1115U	18.8	153.4	1.0	81.5	0.6
	1110U	18.1	98.6	0.9	70.5	2.0
	1166U	17.7	122.7	1.3	72.3	2.0
Pre-symptomatic	1102/18 L	24.3	173.5	2.5	95.7	2.8
	1102/18 R	24.3			93.7	3.0
	1124/18 L	24.0	151.2	4.1	70.8	1.3
	1124/18 R	24.0	149.4	3.0	96.1	1.3
	1128/18 L	24.0	143.0	1.2	56.1	0.5
	1128/18 R	24.0	146.4	13.7	87.5	1.3
Early symptomatic	1151/18 L	24.0	148.5	1.8	61.9	0.9
	1151/18 R	24.0	129.0	6.2	62.6	0.5
	1154/18 L	24.0	163.7	3.7	86.6	2.6
	1154/18 R	24.0	154.0	1.6	77.6	0.6
	1157/18 L	23.8	173.0	6.6	102.3	1.6
	1157/18R	23.8	172.8	6.4	108.7	2.0
Advanced symptomatic	1131/18 L	24.1	160.9	6.5	96.2	0.9
	1131/18 R	24.1	171.5	7.0	97.2	0.9
	1138/18 L	24.0	175.9	3.3	72.0	1.1
	1138/18 R	24.0	124.1	5.5	78.4	1.2
	1170/18 L	23.5	202.4	7.3	100.4	1.5
	1170/18 R	23.5	155.5	5.6	93.0	1.3

Abbreviations: m months; L left treated eye; R right untreated eye

Supplementary Table 5. Outer nuclear layer (ONL) counts following ICV/IVT treatment with scAAV9/oCLN5. Mean outer nuclear cell counts (± SEM) for $CLN5^{-/-}$ sheep treated intracerebroventricularly and intravitreally with scAAV9/oCLN5 at 3 (pre-symptomatic), 6 (early symptomatic), or 9 (advanced-symptomatic) months of age were compared to healthy control $CLN5^{+/-}$ and untreated $CLN5^{-/-}$ sheep. The number of photoreceptor nuclei were counted in at least 20 vertical columns of the retina per animal on hematoxylin and eosin-stained sections.

**Treatment**	**Sheep**	**Age at death (m)**	**Row number**	**SEM**
Healthy control $CLN5^{+/-}$	15-4C	24.0	12.8	0.4
	15-2C	24.0	11.2	0.4
	205-4C	24.0	10.0	0.3
	205-2C	24.0	12.4	0.4
Untreated $CLN5^{-/-}$	1114U	18.3	2.2	0.1
	1115U	18.8	3.2	0.1
	1110U	18.1	1.7	0.1
Pre-symptomatic	1102/18 L	24.3	6.0	0.5
	1102/18 R	24.3		
	1124/18 L	24.0	4.2	0.2
	1124/18 R	24.0	4.8	0.3
	1128/18 L	24.0	3.7	0.1
	1128/18 R	24.0	3.7	0.2
Early symptomatic	1151/18 L	24.0	6.1	0.1
	1151/18 R	24.0	2.0	0.2
	1154/18 L	24.0	4.6	0.2
	1154/18 R	24.0	5.8	0.2
	1157/18 L	23.8	7.1	0.2
	1157/18R	23.8	7.1	0.3
Advanced symptomatic	1131/18 L	24.1	5.7	0.2
	1131/18 R	24.1	5.9	0.2
	1138/18 L	24.0	4.5	0.2
	1138/18 R	24.0	4.5	0.2
	1170/18 L	23.5	7.8	0.2
	1170/18 R	23.5	5.7	0.2

Abbreviations: m months; L left treated eye; R right untreated eye

Supplementary Table 6. Astrocytic response following ICV/IVT treatment with scAAV9/oCLN5. Mean percent area of GFAP immunostaining (± SEM) for $CLN5^{-/-}$ sheep treated intracerebroventricularly and intravitreally with scAAV9/oCLN5 at 3 (pre-symptomatic), 6 (early symptomatic), or 9 (advanced-symptomatic) months of age were compared to healthy control $CLN5^{+/-}$ and untreated $CLN5^{-/-}$ sheep. At least 10 percentage area measurements were taken per hemisphere per animal through three different cortical regions on GFAP-immunostained sections.

**Treatment**	**Sheep**	**Age (m)**	**Visual cortex**		**Parieto-occipital cortex**		**Motor cortex**	
			**% area**	**SEM**	**% area**	**SEM**	**% area**	**SEM**
Healthy control $CLN5^{+/-}$	39C	24.2	2.8	0.3	4.7	0.4	1.7	0.3
	1103C	24.0	2.4	0.2	4.6	0.8	1.7	0.6
	1111C	24.1	2.5	0.2	4.2	0.6	1.8	0.4
Untreated $CLN5^{-/-}$	1104U	23.7	53.9	1.2	56.9	1.6	10.6	1.0
	1109U	23.2	53.4	1.9	53.6	1.8	9.2	1.9
	1116U	23.4	57.6	1.8	59.7	2.0	9.5	2.3
Pre-symptomatic	1102/18	24.3	9.4	1.7	14.5	1.9	1.7	0.2
	1124/18	24.0	12.4	0.9	8.9	1.3	0.5	0.1
	1128/18	24.0	11.0	1.9	33.5	1.2	0.6	0.1
Early symptomatic	1151/18	24.0	18.5	1.9	26.2	3.3	1.3	0.2
	1154/18	24.0	19.2	2.2	35.4	3.6	1.9	0.4
	1157/18	23.8	30.7	2.5	28.3	2.6	2.5	0.2
Advanced symptomatic	1131/18	24.1	16.5	1.8	9.2	0.8	1.6	0.5
	1138/18	24.0	12.7	2.3	34.9	1.9	1.3	0.2
	1170/18	23.5	13.6	1.3	20.7	2.0	3.7	0.9

Abbreviations: m months

Supplementary Table 7. Microglial response following ICV/IVT treatment with scAAV9/oCLN5. Mean percent area of GSB4 staining (± SEM) for CLN5$^{-/-}$ sheep treated intracerebroventricularly and intravitreally with scAAV9/oCLN5 at 3 (pre-symptomatic), 6 (early symptomatic), or 9 (advanced-symptomatic) months of age were compared to healthy control CLN5$^{+/-}$ and untreated CLN5$^{-/-}$ sheep. At least 10 percentage area measurements were taken per hemisphere per animal through three different cortical regions on GSB4-stained sections.

Treatment	Sheep	Age (m)	Visual cortex		Parieto-occipital cortex		Motor cortex	
			% area	SEM	% area	SEM	% area	SEM
Healthy control CLN5$^{+/-}$	39C	24.2	0.00	0.00	0.02	0.00	0.01	0.00
	1103C	24.0	0.02	0.00	0.01	0.01	0.00	0.00
	1111C	24.1	0.01	0.01	0.01	0.00	0.05	0.05
Untreated CLN5$^{-/-}$	1104U	23.7	6.17	0.81	5.95	0.65	3.98	0.55
	1109U	23.2	7.53	0.58	7.34	0.74	4.91	0.43
	1116U	23.4	5.63	0.50	5.14	0.43	3.62	0.13
Pre-symptomatic	1102/18	24.3	2.81	0.33	2.22	0.22	0.02	0.00
	1124/18	24.0	2.30	0.19	0.57	0.07	0.02	0.01
	1128/18	24.0	4.49	0.77	2.73	0.42	0.23	0.05
Early symptomatic	1151/18	24.0	3.28	0.51	1.33	0.17	0.49	0.05
	1154/18	24.0	1.02	0.14	1.62	0.20	0.19	0.07
	1157/18	23.8	0.41	0.10	0.53	0.09	0.02	0.00
Advanced symptomatic	1131/18	24.1	4.79	0.29	1.36	0.11	0.78	0.14
	1138/18	24.0	2.30	0.22	3.31	0.49	0.47	0.12
	1170/18	23.5	2.76	0.30	1.02	0.10	0.24	0.02

Abbreviations: m months

Supplementary Table 8. Lysosomal storage following ICV/IVT treatment with scAAV9/oCLN5. Mean percent area of fluorescence in the brain (± SEM) for $CLN5^{-/-}$ sheep treated intracerebroventricularly and intravitreally with scAAV9/oCLN5 at 3 (pre-symptomatic), 6 (early symptomatic), or 9 (advanced-symptomatic) months of age were compared to healthy control $CLN5^{+/-}$ and untreated $CLN5^{-/-}$ sheep. At least 10 percentage area measurements were taken per hemisphere per animal through three different cortical regions on unstained sections.

**Treatment**	**Sheep**	**Age (m)**	**Visual cortex**		**Parieto-occipital cortex**		**Motor cortex**	
			**% area**	**SEM**	**% area**	**SEM**	**% area**	**SEM**
Healthy control $CLN5^{+/-}$	39C	24.2	0.04	0.01	0.03	0.01	0.02	0.01
	1103C	24.0	0.09	0.02	0.05	0.01	0.15	0.04
	1111C	24.1	0.03	0.01	0.04	0.01	0.04	0.01
Untreated $CLN5^{-/-}$	1104U	23.7	5.35	0.27	4.34	0.88	3.92	0.62
	1109U	23.2	6.31	0.34	6.18	0.40	4.61	0.56
	1116U	23.4	6.13	0.44	6.00	0.28	4.41	0.55
Pre-symptomatic	1102/18	24.3	3.27	0.41	3.06	0.31	2.10	0.20
	1124/18	24.0	2.01	0.31	1.33	0.24	0.97	0.09
	1128/18	24.0	2.47	0.28	4.04	0.74	2.70	0.25
Early symptomatic	1151/18	24.0	2.69	0.49	3.17	0.19	1.79	0.24
	1154/18	24.0	3.10	0.35	2.56	0.26	1.89	0.21
	1157/18	23.8	2.17	0.27	2.13	0.14	1.60	0.20
Advanced symptomatic	1131/18	24.1	3.87	0.35	3.10	0.44	2.59	0.30
	1138/18	24.0	2.13	0.44	3.30	0.39	2.49	0.17
	1170/18	23.5	4.24	0.32	2.07	0.35	2.30	0.17

Abbreviations: m months

Supplementary Table 9. Retinal lysosomal storage following ICV/IVT treatment with scAAV9/oCLN5. Mean percent area of fluorescence in the central retina (± SEM) for $CLN5^{-/-}$ sheep treated intracerebroventricularly and intravitreally with scAAV9/oCLN5 at 3 (pre-symptomatic), 6 (early symptomatic), or 9 (advanced-symptomatic) months of age were compared to healthy control $CLN5^{+/-}$ and untreated $CLN5^{-/-}$ sheep. At least 10 percentage area measurements were taken per retina per animal on unstained sections.

**Treatment**	**Sheep**	**Age at death (m)**	**Central**	
			**% area**	**SEM**
Healthy control $CLN5^{+/-}$	15-4C	24.0	0.2	0.0
	15-2C	24.0	0.2	0.0
	205-4C	24.0	0.1	0.0
	205-2C	24.0	0.1	0.0
Untreated $CLN5^{-/-}$	1114U	18.3	0.2	0.1
	1115U	18.8	0.2	0.0
	1110U	18.1	3.8	1.4
	1166U	17.7	1.0	0.4
Pre-symptomatic	1102/18 L	24.3	1.2	0.2
	1102/18 R	24.3	0.9	0.2
	1124/18 L	24.0	0.2	0.0
	1124/18 R	24.0	0.8	0.1
	1128/18 L	24.0	2.2	0.3
	1128/18 R	24.0	2.0	0.1
Early symptomatic	1151/18 L	24.0	0.7	0.1
	1151/18 R	24.0	2.5	0.2
	1154/18 L	24.0	0.9	0.1
	1154/18 R	24.0	0.9	0.2
	1157/18 L	23.8	0.1	0.0
	1157/18R	23.8	0.4	0.1
Advanced symptomatic	1131/18 L	24.1	0.2	0.1
	1131/18 R	24.1	0.4	0.0
	1138/18 L	24.0	1.0	0.2
	1138/18 R	24.0	1.1	0.1
	1170/18 L	23.5	0.2	0.0
	1170/18 R	23.5	0.4	0.0

Abbreviations: m months; L left treated eye; R right untreated eye

Supplementary Table 10. Mean rate [95% CI] of ERG amplitude decline (µV/month) following ICV/IVT treatment with scAAV9/oCLN5.

**Treatment Group**	**Left treated eye**	**Right untreated eye**
Pre-symptomatic	-12.4 *[-20.73, -3.59]*	-12.8 *[-21.18, -3.36]*
Early symptomatic	-8.5 *[-26.39, 10.55]*	-16.1 *[-34.39, 2.53]*
Advanced symptomatic	-8.5 *[-24.46, 6.04]*	-13.0 *[-29.89, 1.58]*
Control CLN5$^{+/-}$ (*n*=6)	-3.0 *[-6.8, 0.8]*	
Untreated CLN5$^{-/-}$ (*n*=6)	-12.8 *[-16.3, -8.9]*	

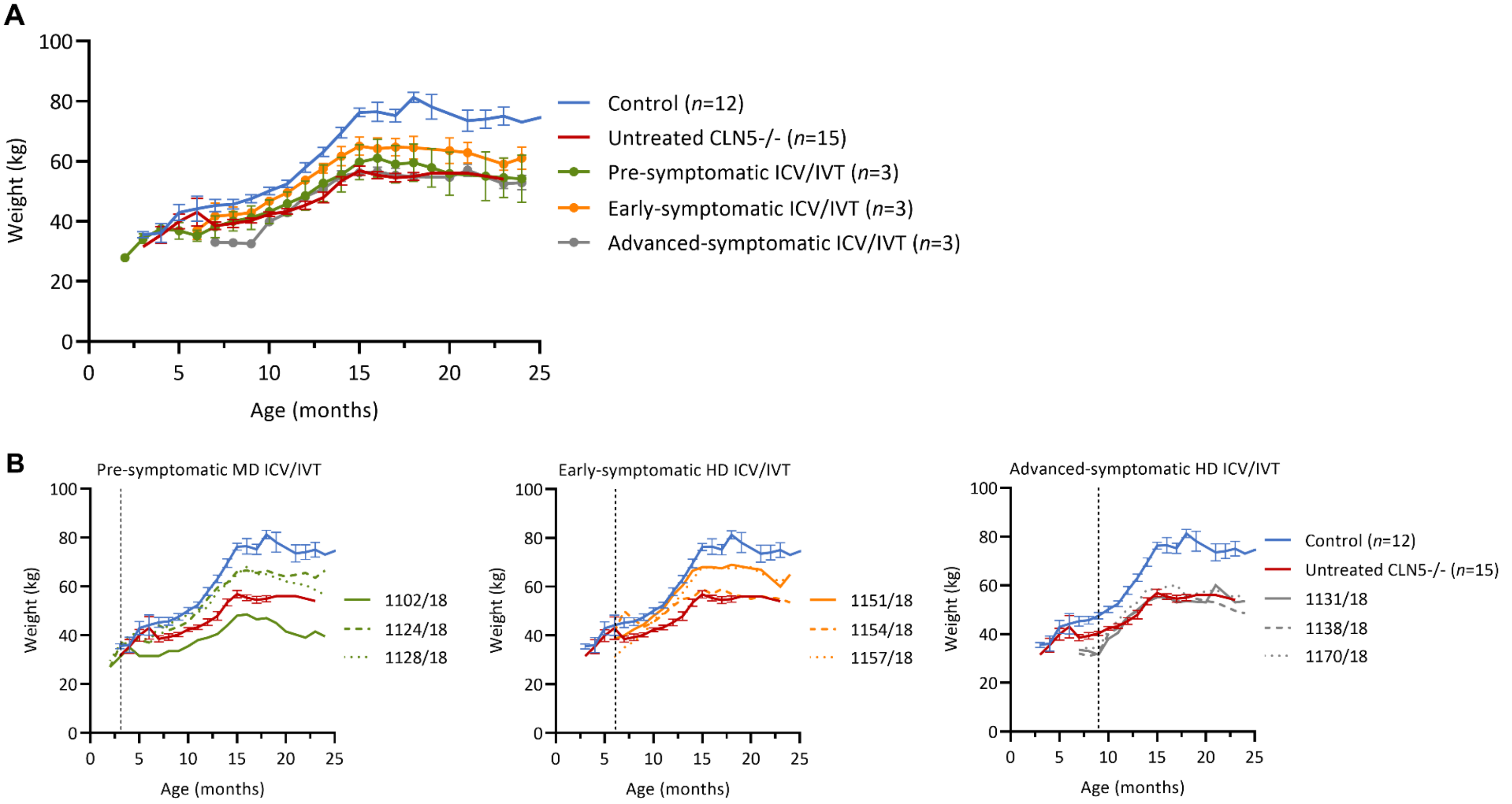

Supplementary Figure 1. Variability in live weight gains following ICV/IVT scAAV9/oCLN5. (A) Average live weight changes (±SEM) in ICV/IVT treated $CLN5^{-/-}$ sheep were compared to average data from healthy control $CLN5^{+/-}$ (blue) and untreated $CLN5^{-/-}$ sheep (red). Control data (*n*=3-4) was collected concurrently with this study and pooled with historical controls for presentation. Pre-symptomatic and early symptomatic treated sheep gained the most weight over their lifetime, whilst advanced symptomatic sheep remained in the range of untreated $CLN5^{-/-}$ sheep. B. Individual live weight changes are displayed by treatment group (pre-symptomatic, green; early symptomatic, orange; advanced symptomatic, grey) at moderate (MD) or high (HD) doses. Dashed lines indicate treatment age.

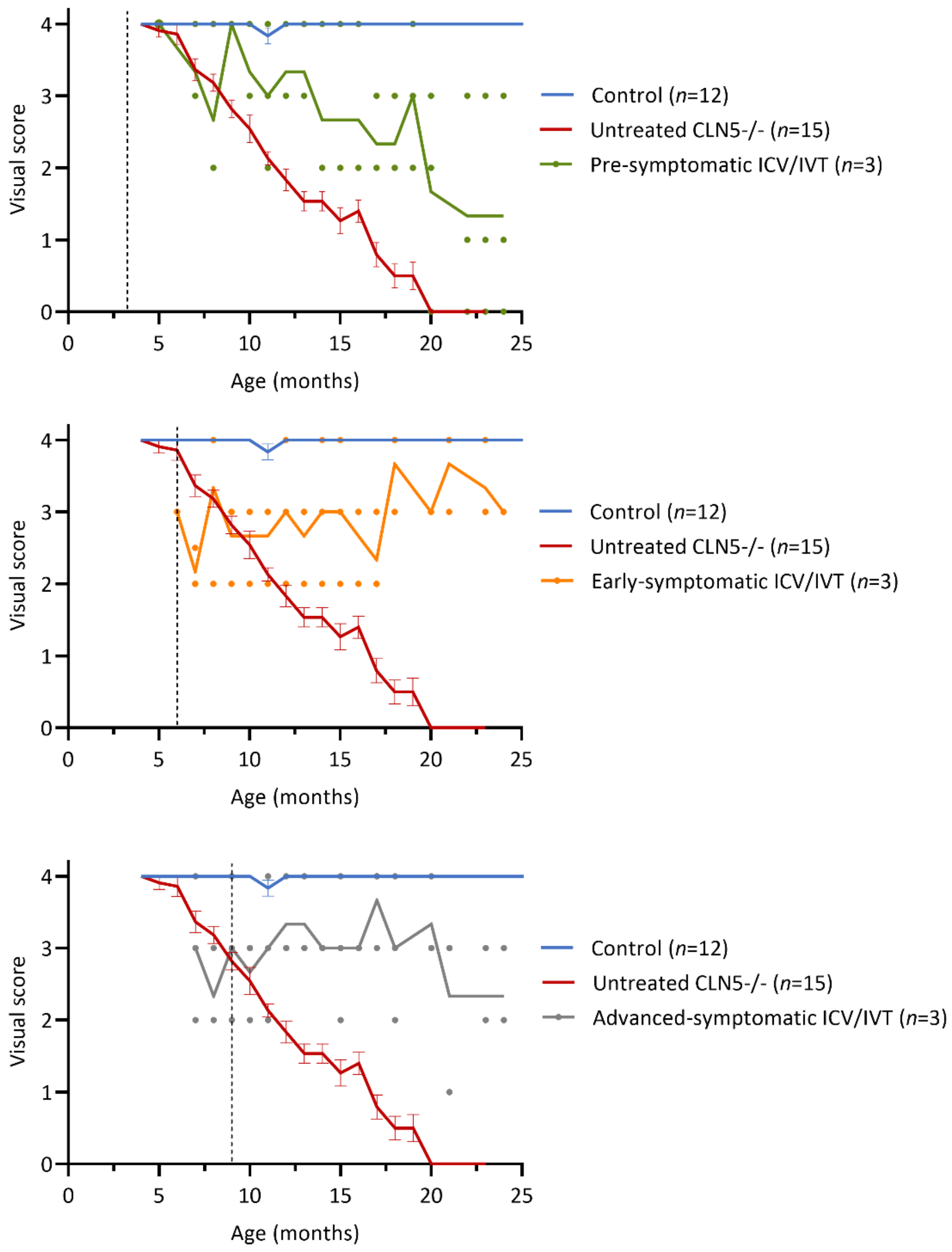

Supplementary Figure 2. High dose ICV/IVT scAAV9/oCLN5 sustains visual function. Vision was maintained to 24 months of age in animals treated at symptomatic disease stages with a high ICV/IVT dose. In comparison, pre-symptomatic treated sheep received an identical IVT dose but a ten-fold lower ICV dose, and two of three sheep in this cohort lost their vision. Individual (dots) and average (line) scores for the visual domain of the oBDRS in ICV/IVT treated $CLN5^{-/-}$ sheep were compared to average historic and concurrent data (±SEM) from healthy control $CLN5^{+/-}$ (blue) and untreated $CLN5^{-/-}$ sheep (red). Dashed lines indicate treatment age.

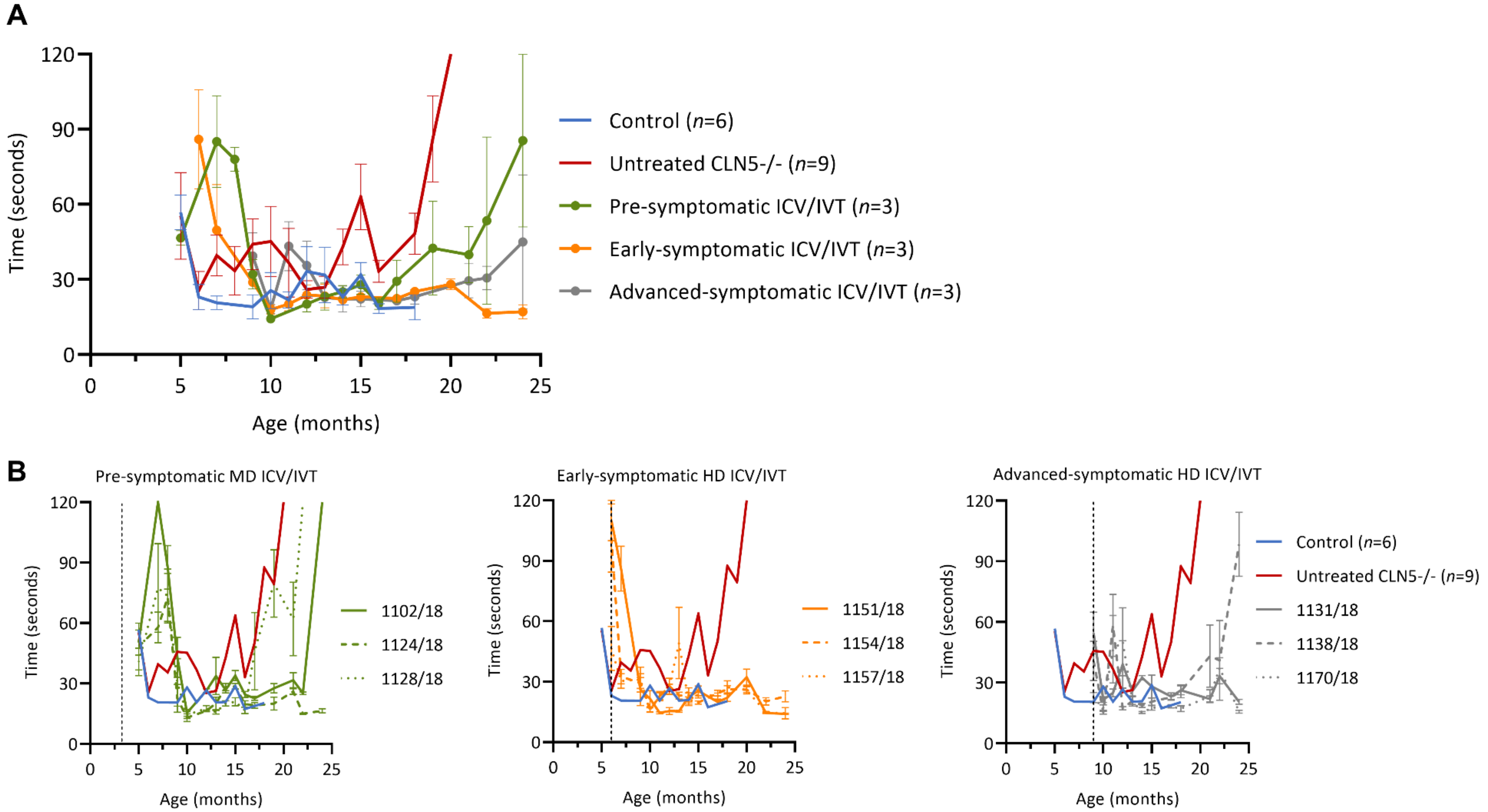

Supplementary Figure 3. High dose ICV/IVT scAAV9/oCLN5 sustains cognition and visual function. (A) Greater long-term maze functionality was afforded to sheep who received the high dose ICV/IVT treatment. Average maze traverse times (±SEM) for ICV/IVT treated CLN5$^{-/-}$ sheep were compared to average data from age-matched healthy control CLN5$^{+/-}$ (blue) and untreated CLN5$^{-/-}$ sheep (red). (B) Individual maze times are displayed by treatment group group (pre-symptomatic, green; early symptomatic, orange; advanced symptomatic, grey) at moderate (MD) or high (HD) doses. Dashed lines indicate treatment age.

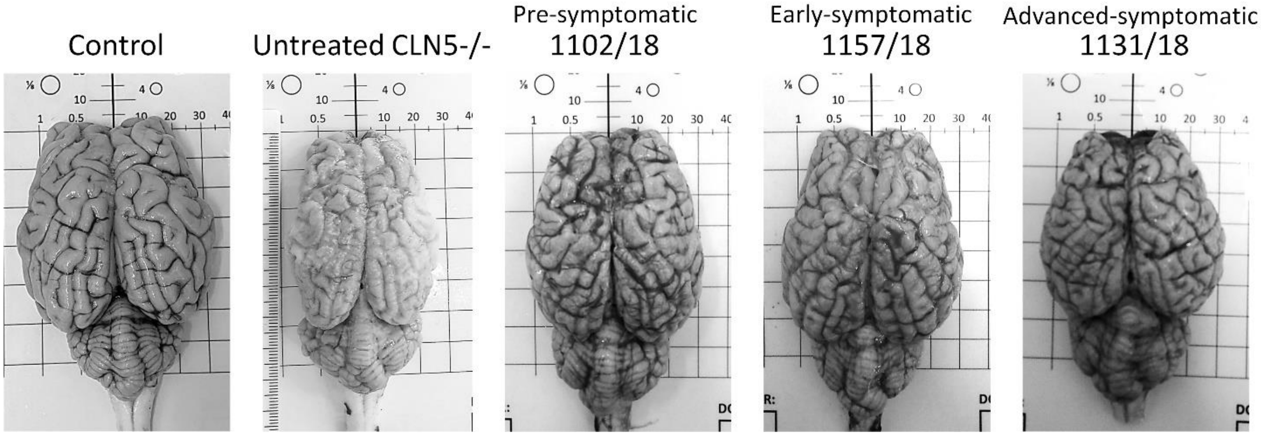

Supplementary Figure 4. ICV/IVT scAAV9/oCLN5 preserves gross brain anatomy. Representative images of brains from 24-month-old $CLN5^{-/-}$ sheep who were treated intracerebroventricularly and intravitreally with scAAV9/oCLN5 at 3 (pre-symptomatic), 6 (early symptomatic), or 9 (advanced symptomatic) months of age compared to age-matched healthy control $CLN5^{+/-}$ and untreated $CLN5^{-/-}$ sheep. Treated brains were larger and had less gyral flattening than age-matched untreated $CLN5^{-/-}$ brains. Markings on the grid to the left of the brain are in inches and to the right are in millimetres.

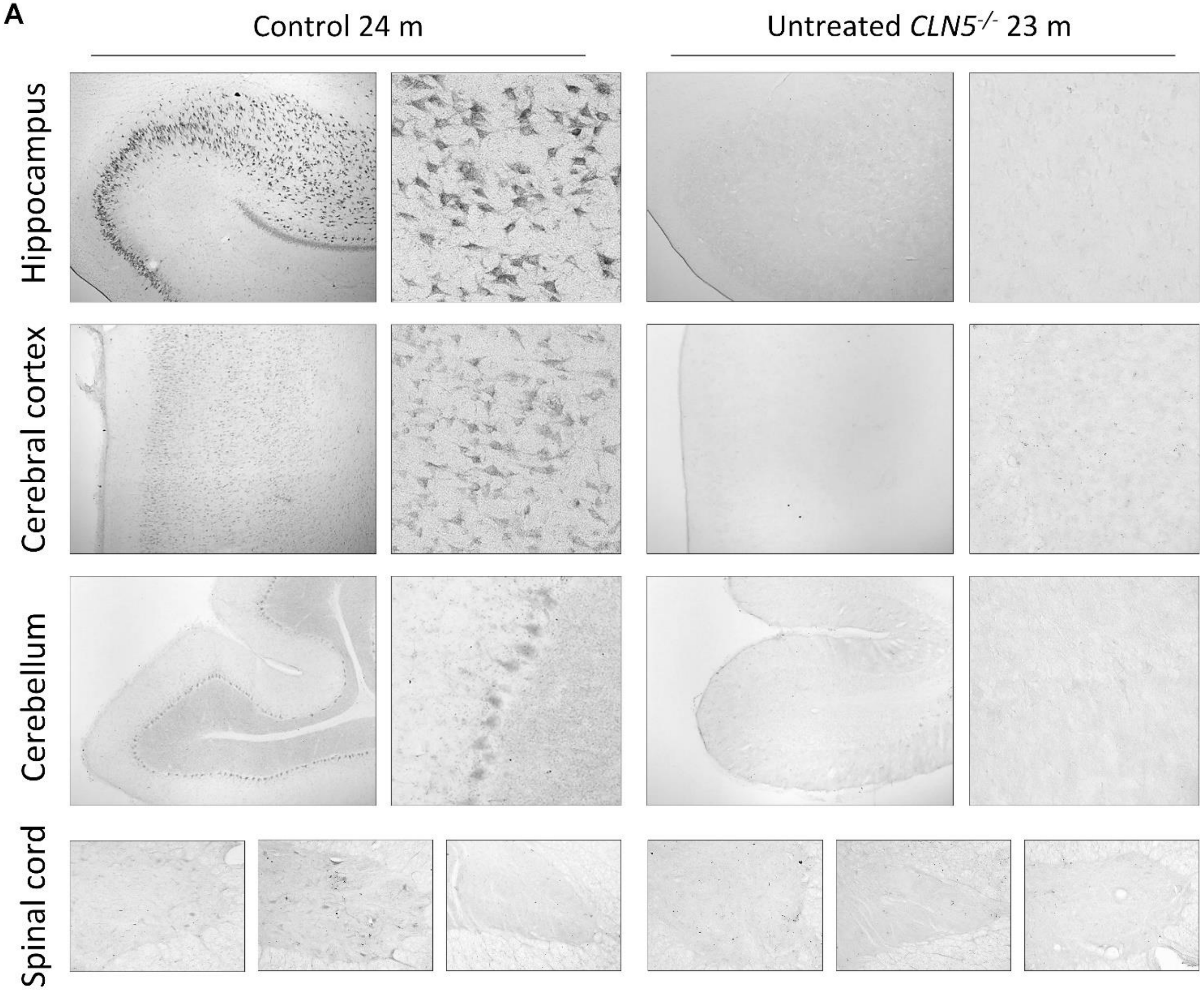
A
Control 24 m
Untreated CLN5-/- 23 m
Hippocampus
Cerebral cortex
Cerebellum
Spinal cord

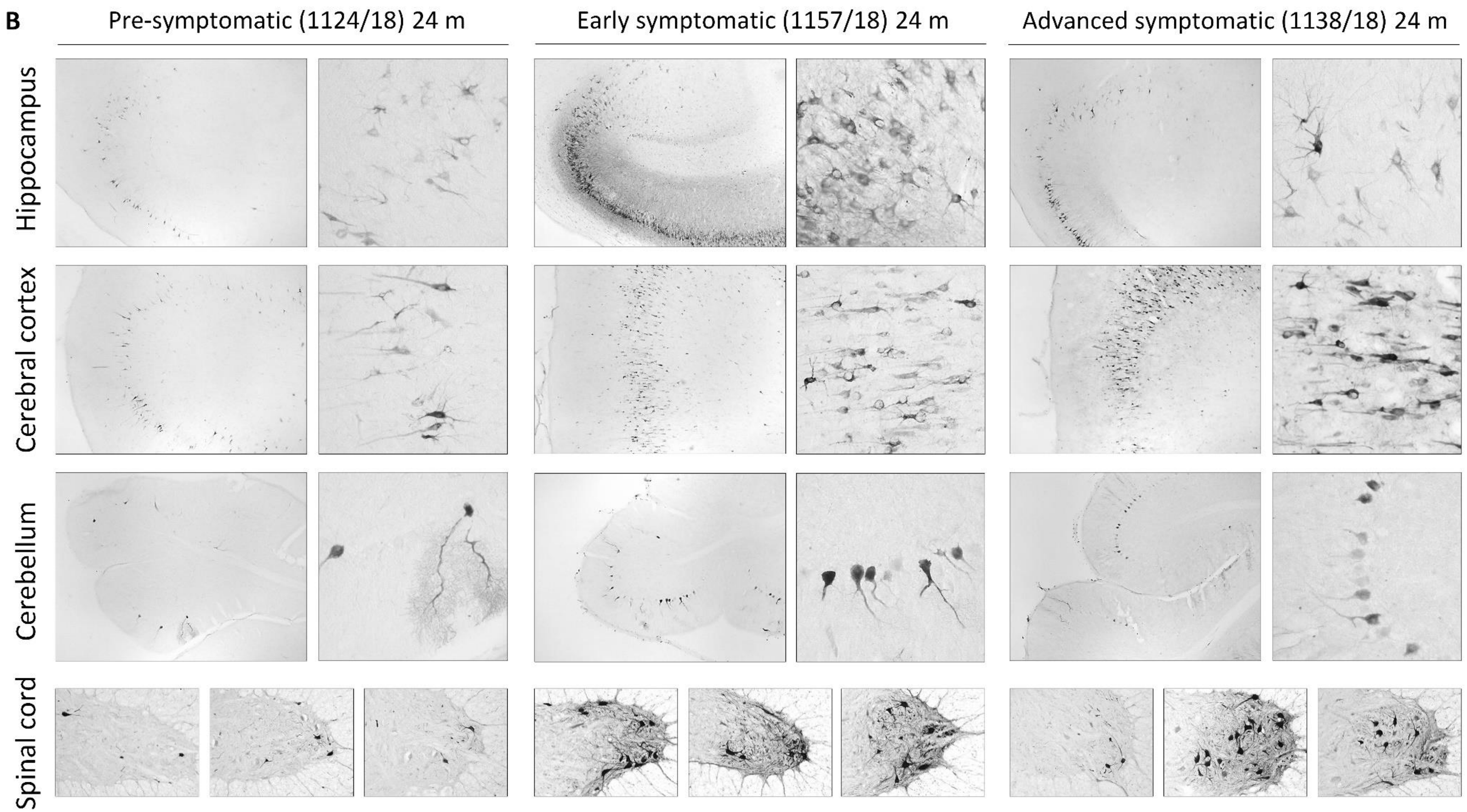
B
Pre-symptomatic (1124/18) 24 m
Early symptomatic (1157/18) 24 m
Advanced symptomatic (1138/18) 24 m
Hippocampus
Cerebral cortex
Cerebellum
Spinal cord

Supplementary Figure 5. Ovine CLN5 protein is expressed in the brain and spinal cord of ICV/IVT scAAV9/oCLN5 treated sheep. (A) Representative CLN5 immunostaining in the central nervous system from healthy control $CLN5^{+/-}$ and untreated $CLN5^{-/-}$ sheep. Endogenous CLN5 protein expression was evident throughout the healthy control $CLN5^{+/-}$ brain, particularly in the hippocampus, cortical neurons and cerebellar Purkinje cells whilst there was no CLN5 expression in the untreated $CLN5^{-/-}$ brain. (B) Representative CLN5 immunostaining in the same regions from 24-month-old $CLN5^{-/-}$ sheep who were treated intracerebroventricularly and intravitreally with scAAV9/oCLN5 at 3 (pre-symptomatic), 6 (early symptomatic), or 9 (advanced symptomatic) months of age show extensive CNS transduction. Left to right: cervical, thoracic and lumbar spinal cord ventral horn images.

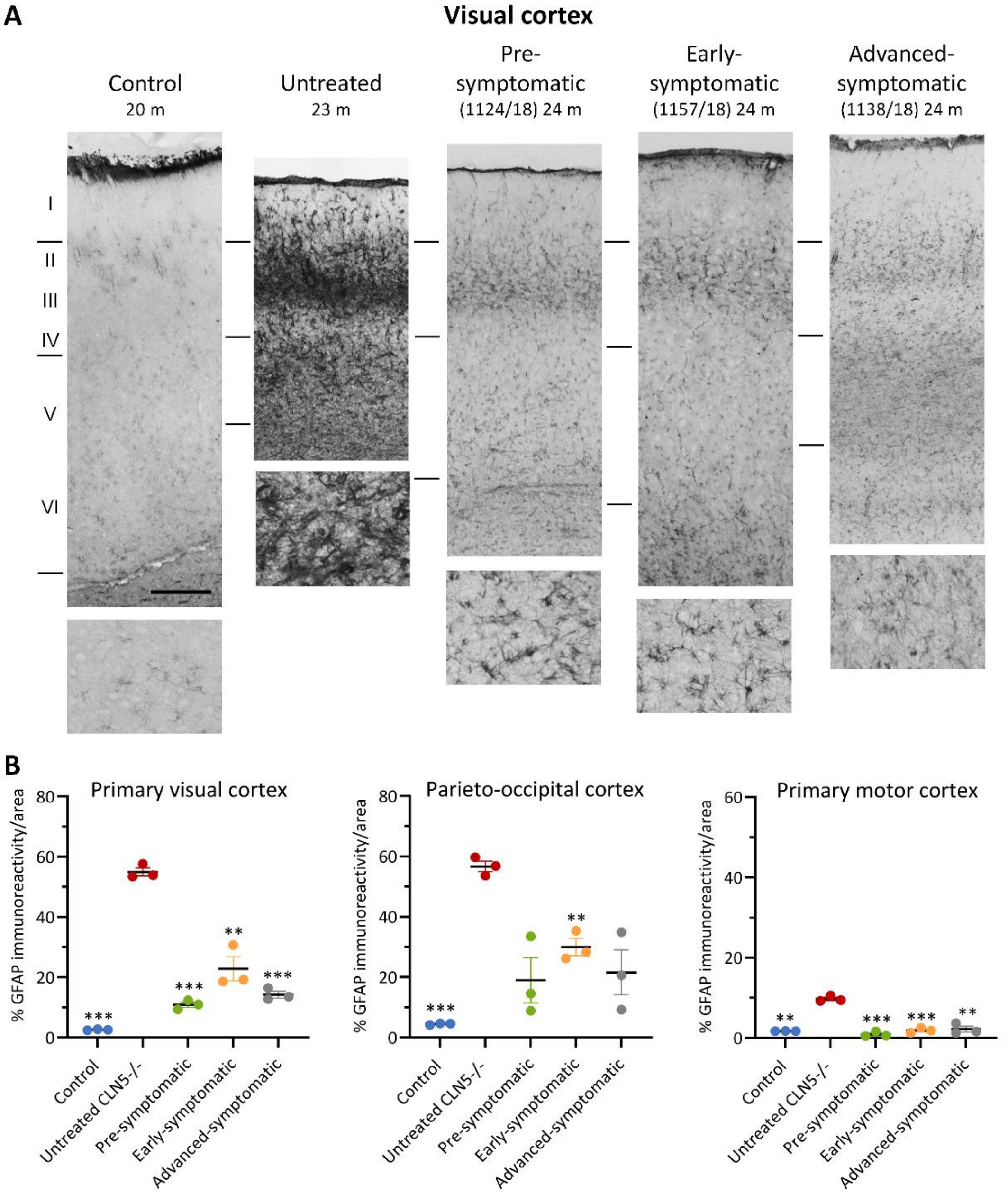

Supplementary Figure 6. ICV/IVT scAAV9/oCLN5 attenuates astrocytosis. (A) The positive effect on astroglial activation can be seen in representative GFAP-immunostained images from the visual cortex of 24-month-old $CLN5^{-/-}$ sheep who were treated intracerebroventricularly and intravitreally with scAAV9/oCLN5 at 3 (pre-symptomatic, green), 6 (early symptomatic, orange), or 9 (advanced symptomatic, grey) months of age when compared to healthy control $CLN5^{+/-}$ and untreated $CLN5^{-/-}$ sheep. (B) Quantification of astrocytosis in three key regions which differentially undergo neurodegeneration in ovine CLN5 disease shows the treatment effect on individual animals. Vertical bars denote ± SEM. Significant differences to untreated $CLN5^{-/-}$ are denoted by asterisks (*** $P < 0.001$, ** $P < 0.01$).

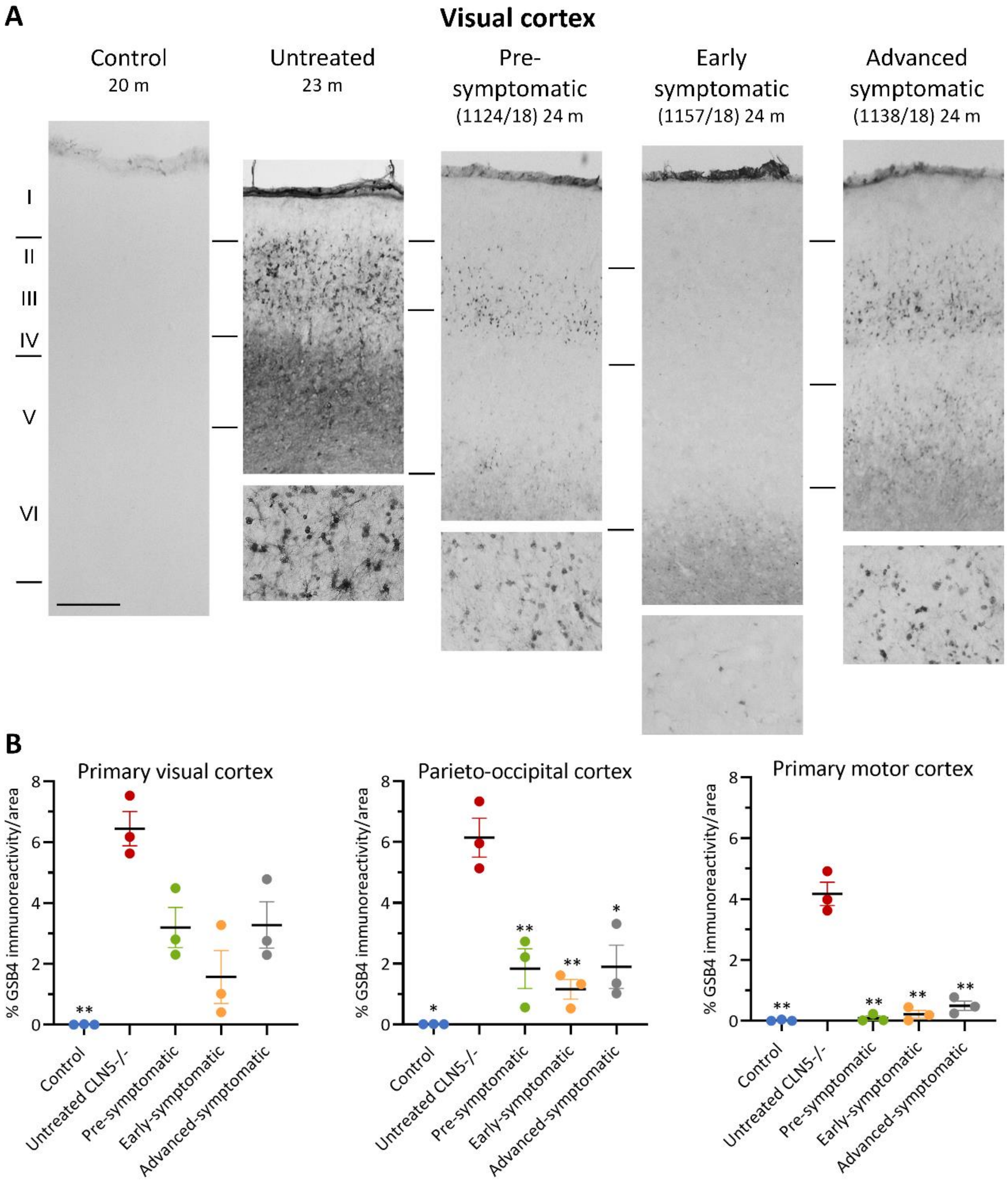

Supplementary Figure 7. ICV/IVT scAAV9/oCLN5 attenuates microgliosis. (A) The positive effect on microglial activation can be seen in representative GSB4-stained images from the visual cortex of 24-month-old $CLN5^{-/-}$ sheep who were treated intracerebroventricularly and intravitreally with scAAV9/oCLN5 at 3 (pre-symptomatic), 6 (early symptomatic), or 9 (advanced symptomatic) months of age compared to healthy control $CLN5^{+/-}$ and untreated $CLN5^{-/-}$ sheep. (B) Quantification of microgliosis in three key regions which differentially undergo neurodegeneration in ovine CLN5 disease shows the treatment effect on individual animals. Vertical bars denote ± SEM. Significant differences to untreated $CLN5^{-/-}$ are denoted by asterisks (** $P < 0.01$).

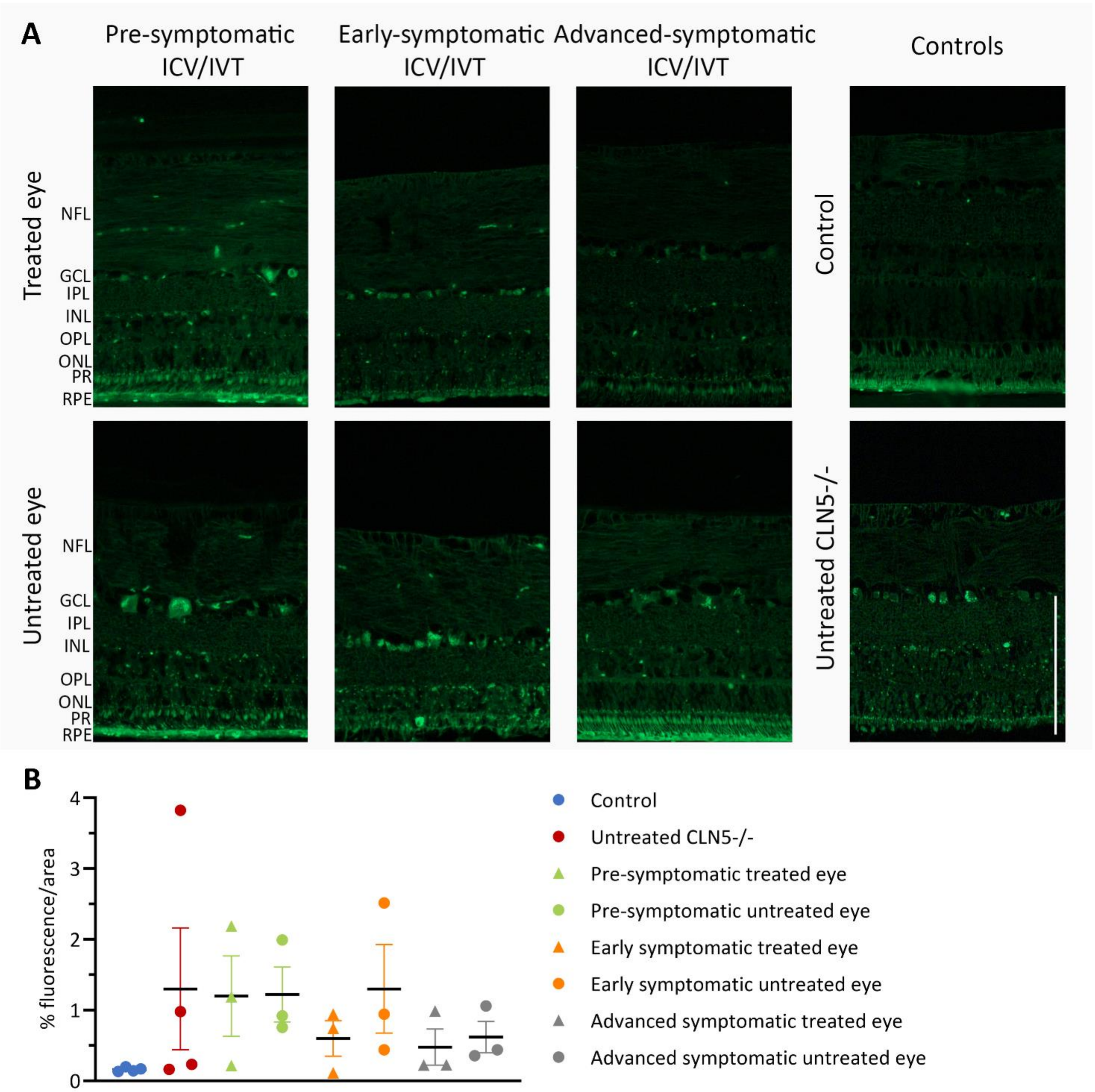

Supplementary Figure 8. ICV/IVT scAAV9/oCLN5 had a variable effect on retinal lysosomal storage. (A) Representative retinal sections displaying fluorescent lysosomal storage material from the treated and untreated eye of 24-month-old ICV/IVT treated CLN5$^{-/-}$ sheep compared to age-matched healthy control CLN5$^{+/-}$ and untreated CLN5$^{-/-}$ retina. NFL, nerve fiber layer; GCL, ganglion cell layer; IPL, inner plexiform layer; INL, inner nuclear layer; OPL, outer plexiform layer; ONL, outer nuclear layer; PR, photoreceptor layer; RPE, retinal pigment epithelium. Scale bar (in lower right image) represents 100µm. (B) Whilst there was a trend towards lower levels of storage burden (mean percentage area of fluorescence) in treated eyes, quantification of storage burden was not statistically different between treated and untreated eyes. Vertical bars denote ± SEM.
